# Regulating Chlorine and Hydrogen Atom Transfer for Selective Photoelectrochemical C─C Coupling by Cu‐coordination Effect at Semiconductor/Electrolyte Interfaces

**DOI:** 10.1002/advs.202408767

**Published:** 2024-10-24

**Authors:** Qiaozhen Li, Kun Dang, Lei Wu, Siqin Liu, Yuchao Zhang, Jincai Zhao

**Affiliations:** ^1^ Key Laboratory of Photochemistry CAS Research/Education Center for Excellence in Molecular Sciences Institute of Chemistry Chinese Academy of Sciences Beijing 100190 P. R. China; ^2^ University of Chinese Academy of Sciences Beijing 100049 P. R. China

**Keywords:** chlorine, copper, hydrogen atom transfer, Minisci reaction, photoelectrochemistry

## Abstract

Semiconductor‐based photoelectrochemical (PEC) organic transformations usually show radical characteristics, in which the reaction selectivity is often difficult to precisely control due to the nonselectivity of radicals. Accordingly, several simple organic reactions (e.g., oxidations of alcohols, aldehydes, and other small molecules) have been widely studied, while more complicated processes like C─C coupling remain challenging. Herein, a synergistic heterogeneous/homogeneous PEC strategy is developed to achieve a controllable radical‐induced C─C coupling reaction mediated by the copper‐coordination effect at the semiconductor/electrolyte interfaces, which additionally exerts a significant impact on the product regioselectivity. Through experimental studies and theoretical simulations, this study reveals that the copper‐chloride complex effectively regulates the formation of chloride radicals, a typical hydrogen atom transfer agent, on semiconductor surfaces and stabilizes the heterogeneous interfaces by suppressing the radical‐induced surface passivation. Taking the Minisci reaction (the coupling between 2‐phenylquinoline and cyclohexane) as a model, the yield of the target C─C coupling product reaches up to 90% on TiO_2_ photoanodes with a selectivity of 95% and long‐term stability over 100 h. Moreover, such a strategy exhibits a broad scope and can be used for the functionalization of various heteroaromatic hydrocarbons.

## Graphical abstract

A synergistic heterogeneous/homogeneous PEC strategy mediated by Cu^2+^ achieves a controllable radical‐induced C─C coupling reaction between aliphatic compounds with inert C(sp^3^)─H bond and a wide range of aromatics by using TiO_2_ photoanodes. Experimental studies and theoretical simulations reveal that the presence of Cu^2+^ regulates the formation of chloride radicals and stabilizes the heterogeneous interfaces.

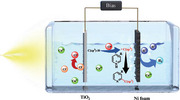

## 1 Introduction

Semiconductor‐based photoelectrochemical (PEC) water splitting reaction has been widely studied for hydrogen production,^[^ ^1^, ^2^, ^3^, ^4^ ^]^ but the overall performance is limited by the anodic oxygen evolution reaction (OER) with sluggish kinetics.^[^ ^5^, ^6^, ^7^, ^8^, ^9^, ^10^ ^]^ Alternatively, PEC organic reactions,^[^ ^8^, ^11^, ^12^ ^]^ such as alcohol oxidation,^[^ ^13^, ^14^, ^15^, ^16^ ^]^ have drawn much attention due to their more favorable thermodynamics and the production of high‐valued chemicals. For example, glycerol is oxidized to 1,3‐dihydroxyacetone by nanoporous BiVO_4_ photoanodes in an acidic medium without the addition of oxidizers.^[^ ^15^ ^]^ Similarly, the high‐performance PEC oxidation of 5‐hydroxymethylfurfural (HFM) is also reported.^[^ ^17^ ^]^ In addition to simple oxidation, PEC activation of C─H bonds provides more possibilities for artificial photosynthesis.^[^ ^18^, ^19^, ^20^, ^21^ ^]^ For example, cyclohexanol and cyclohexanone are prepared by the C─H activation of cyclohexane, which enables the construction of C─O bonds and achieves high oxidation selectivity.^[^ ^18^ ^]^ The reaction of electron‐rich aromatic hydrocarbons with azole compounds to produce medicinal nitrogen heterocycles has been realized by a PEC method.^[^ ^20^ ^]^ The construction of the C─P bond has also been realized, which shows good functional group tolerance.^[^ ^21^ ^]^

Even though photoelectrochemistry has achieved the construction of C─P bonds and C─N bonds, these works only account for a tiny part of organic reactions. The reported semiconductor‐based PEC organic transformations usually show radical characteristics, and the reaction selectivity is often difficult to precisely control due to the nonselectivity of radicals. Therefore, developing new PEC strategies to achieve more complex reactions (e.g., C─C coupling) with high product selectivity still needs to be explored.^[^ ^22^, ^23^, ^24^ ^]^ It is a feasible way to construct C─C bonds by directly forming alkane radicals with C(sp^3^)─H activation, which can be expanded to a wide range of substrates.^[^ ^25^ ^]^ However, as one of the most prevalent but least active bonds in organic molecules, it is challenging to break the C(sp^3^)─H bond due to the high bond dissociation energies (413 kJ mol^−1^).^[^ ^26^, ^27^ ^]^

Semiconductor photoelectrodes with deep valence bands, such as TiO_2_, can directly oxidize chlorine ion (Cl^−^) to obtain chlorine radical (Cl**^·^**),^[^ ^19^, ^28^ ^]^ an emerging hydrogen atom transfer (HAT) agent for C─H bond cleavage,^[^ ^25^, ^26^, ^27^, ^28^, ^29^, ^30^, ^31^ ^]^ which is promising to generate alkyl radicals for C─C coupling through the HAT mechanism. However, we find that this reaction suffers from poor selectivity and stability. Herein, we develop a synergistic heterogeneous/homogeneous PEC strategy via the incorporation of copper ions (Cu^2+^) for highly selective and stable C─C coupling. Through experimental studies and theoretical calculations, we demonstrate that Cu^2+^ at semiconductor/electrolyte interfaces can effectively regulate the formation of Cl**^·^** on TiO_2_ surfaces and stabilize the heterogeneous interfaces. Interestingly, excellent regioselectivity is achieved owing to the coordination effect of the CuCl_x_ complex. Taking the Minisci radical coupling (the coupling between 2‐phenylquinoline and cyclohexane) as a model reaction, a high C─C coupling product yield of 90% together with a selectivity of 95% and long‐term stability over 100 h are realized. This strategy exhibits a broad spectrum of applications and can be employed to functionalize diverse heteroaromatic hydrocarbons.

## 2 Results and Discussion

**Figure 1:**
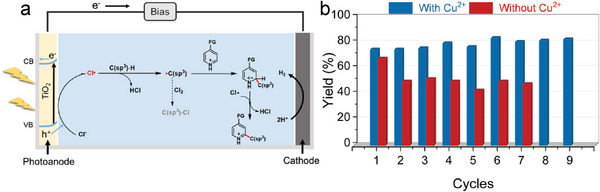
The Minisci radical coupling reaction on a TiO_2_ photoanode. a) Schematic of the PEC process. b) Reaction performance and stability test for the C─C coupling for 91 h in batch reaction (7 batches) on a TiO_2_ photoanode, and with Cu^2+^ for the C─C coupling for 117 h in batch reactions (9 batches). The reaction was carried out in acetone/water solution with 0.1 m TEATFB and 100 mL HCl at 1.2 V_Ag/AgCl_ under the illumination of 380–800 nm (400 mW cm^−2^) with 13 h for every batch. The photoanode was washed by deionized water and acetonitrile after each batch reaction.

The Minisci radical coupling reaction utilizes Cl**^·^** as the HAT reagent to transform alkane into alkane radicals, and then the alkane radicals can be combined with protonated aromatics to obtain C─C coupling products (**Figure** **1**a). The TiO_2_ photoanode can directly oxidize Cl^−^ to obtain Cl**^·^**, which showed a high photovoltage of 1.77 V under 380–800 nm irradiation as the advantage for solar energy utilization (Figure S1, Supporting Information). We screened the conditions for the reaction by using 2‐phenylquinoline (**1A**) and cyclohexane (**2A**) as substrates. Since **1A** itself has an ultraviolet absorption below 380 nm (Figure S2, Supporting Information), the PEC reaction was carried out under light irradiation with wavelength between 380 and 800 nm. 100 µL of HCl was used as the source of Cl^−^ and proton, and 0.1 M of tetraethyl ammonium tetrafluoroborate (TEATFB) was used as the electrolyte (Figure S3, Supporting Information). Under the PEC condition, the yield of **3A** (the target C─C coupling product) reached 70% but only in the first cycle with a freshly prepared photoanode, while the yield decreased to 50% in the second cycle and even lower in the following cycles (Figure 1b). We find that by introducing 5% mol Cu^2+^ into the electrolyte, the yield of **3A** was significantly improved to 80% and showed no decay within 9 cycles, which exhibited a long‐term stability of overall 117 h (Figure 1b). Notably, the color of the TiO_2_ photoanode turned yellow after photoelectrolysis in Cu^2+^‐free solutions when using quinoline as the reaction substrate, while it was not observed for photoanodes exposed to Cu^2+^‐bearing solutions (Figure S4, Supporting Information). Therefore, the presence of Cu^2+^ plays an important role in the stability of TiO_2_. Despite the much‐enhanced C─C coupling behavior, the photocurrent obtained from the linear sweep voltammetry (LSV) measurement showed little difference after introducing Cu^2+^ (Figure S5a, Supporting Information), as well as the steady‐state photocurrent (Figure S5b, Supporting Information).

Through the optimization of reaction conditions, we achieved a high yield of 90% and the conversion rate of 95% in a mixture of acetone and water (V/V = 19/1) containing 5% mol Cu^2+^, 0.1 m TEATFB and 100 µL HCl under 1.6 V_Ag/AgCl_ for 13 h (**Table** **1**, entry 1). As anticipated, the reaction did not proceed in the absence of light or applied bias (Table 1, entry 2–3). The potential‐dependent product yield is presented in Table S1 (Supporting Information) (entry 1–3), in which the product yield significantly increased with higher applied bias. The high light intensity was utilized to enhance the current density, thereby attaining a greater yield within a fixed time (Figure S6, Supporting Information). Lowering the content of Cu^2+^ would decrease the product yield (Table 1, entry 4). On the contrary, increasing the concentration of Cu^2+^ beyond 10% mol led to no improvement in yields (Table 1, entry 5). When the reaction was carried out directly in the air, the yield decreased to 63% (Table 1, entry 6), indicating of the importance to deaerate the reaction electrolyte. Increasing the temperature was conducive to the reaction below 50 °C, whereas an excessive temperature led to the decrease in yield as the solvent started to evaporate (Table S1, Supporting Information; entry 4–5). The absence of TEATFB would result in a significant decrease in the yield of **3A** (Table 1, entry 7), and the yield was also influenced by the type of electrolyte salts (Table S1, Supporting Information; entry 6–9). HCl also plays an important role. It not only provides an acidic environment to protonate **1A**, but also acts as the source of chloride required for HAT (Table 1, entry 8). Therefore, the influence of acid quantities on the yield was investigated, and an optimized content of HCl was found to be 100 µL (Table 1, entry 9–10).

**Table 1:** Optimization of reaction conditions.

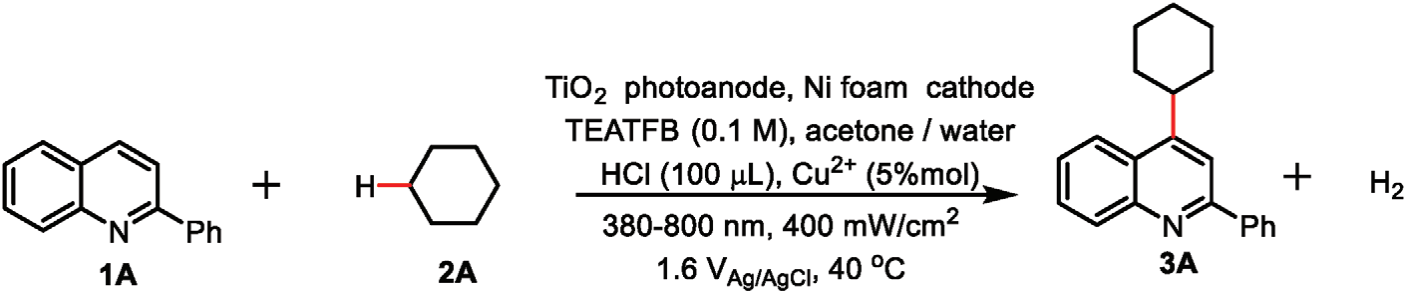				
Entry	HCl [µL]	Cu^2+^ [%mol]	Conversion [%]	Yield [%]^a)^
**1**	100	5	95	90
**2** ^b)^	100	5	0	0
**3** ^c)^	100	5	2	2
**4**	100	1	72	69
**5**	100	10	88	85
**6** ^d)^	100	5	70	63
**7** ^e)^	100	5	33	32
**8**	–	5	0	0
**9**	50	5	68	66
**10**	150	5	78	75

Standard reaction conditions: TiO_2_ as the photoanode, Ni foam as the cathode, 1A (0.1 mmol), 2A (0.3 mL), 5 mL acetone/water (V/V) = 19/1, HCl (100 µL), Cu^2+^ (5% mol), TEATFB (0.1 M), 40 °C, 13 h.

^a)^ Determined by ^1^H NMR analysis using 1,3,5‐trimethoxybenzene as the internal standard;

^b)^ In the dark;

^c)^ No bias;

^d)^ In the air;

^e)^ No electrolyte.

**Figure 2:**
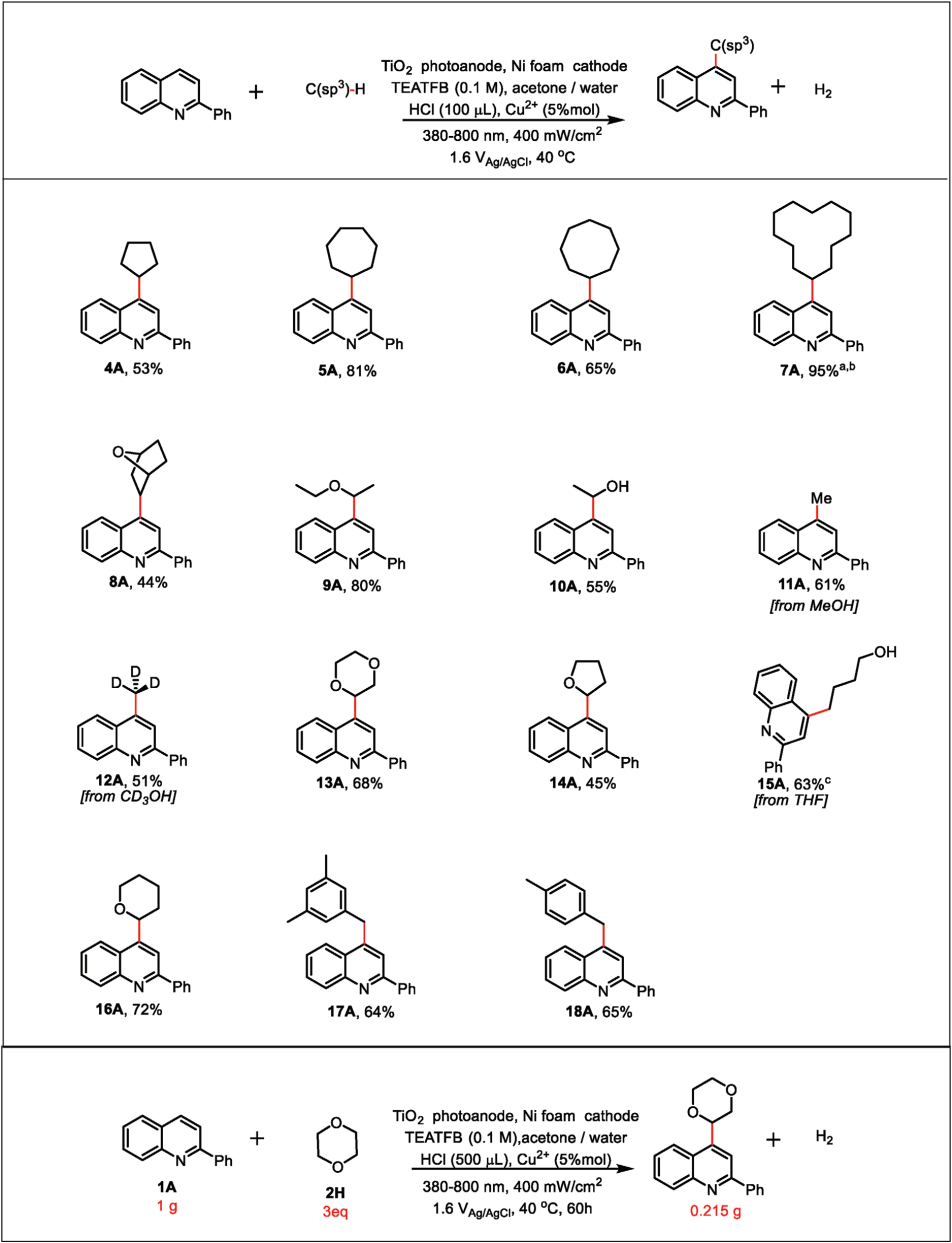
Substrate scope. Reaction conditions: TiO_2_ as the photoanode, Ni foam as the cathode, **1** (0.1 mmol), **2** (0.3 mL for liquid, or 10 equiv. for solid), 5 mL acetone/water (V/V) = 19:1, HCl (100 µL), Cu^2+^ (5% mol), TEATFB (0.1 M), 40 °C, 13 h. Determined by ^1^H NMR analysis using vinyl carbonate as the internal standard. [a] The reaction was run for 24 h. [b] The reaction was heated to 50 °C. [c] 25 µL HCl was used.

To demonstrate the general applicability of the PEC strategy for C─C coupling reaction, we extended the optimal conditions (Table 1, entry 1) to more substrates (**Figure** **2**). We investigated the range of alkanes capable of reacting with **1A**. Simple cycloalkanes similar to cyclohexane afforded a good yield under these conditions (**4A**–**6A**). The low yield of cyclopentane may be attributed to its low boiling point, which was easy to volatilize under these conditions. Cyclododecane afforded the desired product with 95% yield (**7A**) by raising the temperature to 50 °C and prolonging the reaction time to 24 h. Notably, 1,4‐epoxycyclohexane provided heteroarenes (**8A**) by reacting with **1A**. Inspired by this, we explored the reactivity of oxygen‐containing substances. To our gratification, this method was found to be broadly compatible with ethers (**9A**), alcohols (**10A**–**12A**), and epoxides (**13A**–**16A**) and showed a satisfactory yield. However, the C─O cleavage was observed for the reactions of methanol under the standard reaction conditions. In particular, the reaction with tetrahydrofuran (THF) induced the C─O cleavage by decreasing the acid amount (**15A**), while the standard reaction conditions resulted in the C─H bond cleavage (**14A**). It was worth noting that our system can facilitate the reaction between aromatic compounds with a yield of ≈65% (**17A**–**18A**). We carried out a high‐dose experiment by using 4.88 mmol of 2‐phenylquinoline to react with 1,4‐dioxane for 60 h, and obtained 0.215 g of the product by separation.

**Figure 3:**
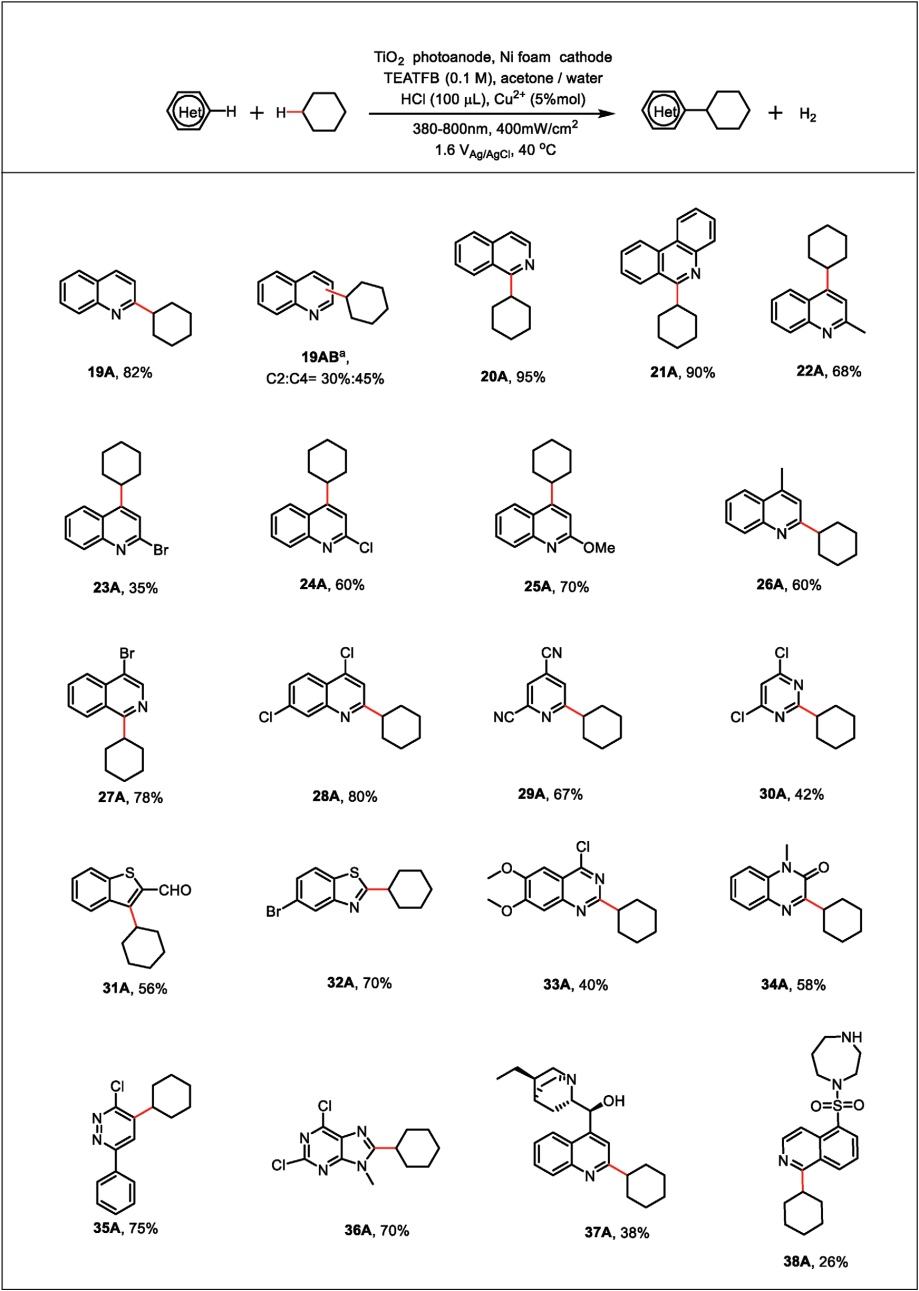
Substrate scope. Reaction conditions: TiO_2_ as the photoanode, Ni foam as the cathode, **1** (0.1 mmol), **2** (0.3 mL for liquid, or 10 equiv. for solid), 5 mL acetone/water (V/V) = 19:1, HCl (100 µL), Cu^2+^ (5% mol), TEATFB (0.1 M), 40 °C, 13 h. Determined by ^1^H NMR analysis using vinyl carbonate as the internal standard. [a] In standard reaction conditions without Cu^2+^.

The substrate and functional group tolerance were further investigated by coupling various heterocycles with **2A** (**Figure** **3**). We were pleased to see that very high regioselectivity was obtained under the PEC conditions. Quinoline was predominantly alkylated at the C2‐position (**19A**).^[^ ^29^, ^30^ ^]^ However, the absence of Cu^2+^ in the solution resulted in poor regioselectivity of products, with a yield of only 30% for the C2‐position, in addition to the presence of the C4‐position (Figure S7, Supporting Information). Moreover, alkylation at the C1‐position resulted in excellent yields for isoquinolines (**20A**) and phenanthridine (**21A**). When quinoline substituted at C2‐position was replaced by methyl, chlorine, bromine functional groups, or methoxy groups, they afforded the corresponding alkylated products with a satisfactory yield at the C4‐position (**22A**–**25A**). Meanwhile, when the substituent group was an electron‐withdrawing group, the yield decreased. On the other hand, quinolones bearing a C4‐substituent underwent alkylation at the C2‐position (**26A**–**28A**). In addition, cyclohexane also underwent reactions with pyridine (**29A**), pyrimidine (**30A**), benzothiophene (**31A**), benzothiazole (**32A**), benzopyrimidine (**33A**), quinoxaline (**34A**), pyridazine (**35A**), and purine (**36A**) aromatics. The PEC strategy was further applied to complex substrates such as hydrocinchonine (**37A**) and Fasudil (**38A**).

**Figure 4:**
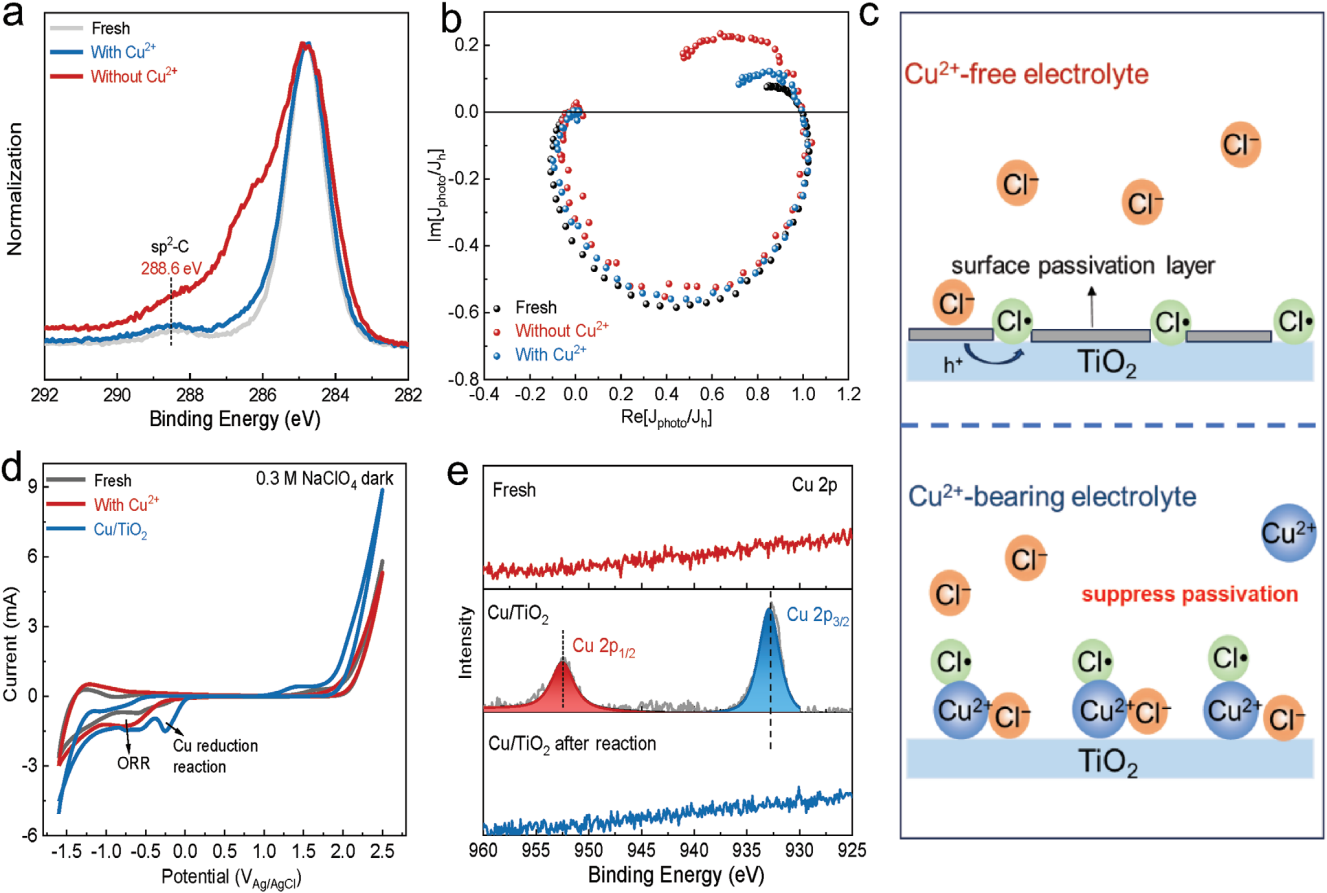
Effect of Cu^2+^ on the TiO_2_ photoanode. a) Normalized C1s XPS spectra of the fresh TiO_2_ (Fresh), the TiO_2_ after reaction without Cu^2+^ and with Cu^2+^. b) Normalized IMPS spectra of the fresh TiO_2_ (Fresh), the TiO_2_ after reaction without Cu^2+,^ and the TiO_2_ after reaction with Cu^2+^. All were performed in standard reaction conditions without Cu^2+^. c) Schematics of TiO_2_ surface in the Cu^2+^‐free electrolyte and Cu^2+^‐bearing electrolyte. d) CVs obtained in 0.3 m NaClO_4_ by using the fresh TiO_2_ (Fresh), the TiO_2_ after reaction in the solution with Cu^2+,^ and the Cu‐modified TiO_2_ (Cu/TiO_2_). The scan rate was 10 mV·s^−1^. e) Cu 2p XPS spectra of the fresh TiO_2_ (Fresh), the Cu/TiO_2_ before and after reactions.

The role of Cu^2+^ is twofold as unveiled in Figure 1b, as it not only maintains the stability of TiO_2_ photoanodes but also enhances the C─C coupling performance. To comprehend these dual functions, we initially assessed its impact on the physical structure of the photoanode. Scanning electron microscopy (SEM) measurements (Figure S8, Supporting Information) and powder X‐ray diffraction (XRD) measurements (Figure S9, Supporting Information) were used to characterize the surface morphology and crystal phase of photoanodes, respectively. Both the SEM and XRD results showed that the structural integrity of the TiO_2_ photoanode remained unaffected when operated in Cu^2+^‐free solutions, although it presented a remarkably decreased performance for the C−C coupling reaction (Figure 1b). The decreased stability of the TiO_2_ photoanode derives from the surface passivation, as revealed by X‐ray photoelectron spectroscopy (XPS) measurement (**Figure** **4**a). The peak intensity of the sp^2^‐C (288.6 eV) displayed an obvious increase for the TiO_2_ photoanode used in the Cu^2+^‐free electrolyte when compared with that of other photoanodes. Accordingly, we suspect that the organics in the Cu^2+^‐free electrolyte may deposit on the photoanode surface during reactions, which resulted in the passivation of the active sites on TiO_2_ surfaces. Fortunately, such a process can be effectively suppressed by introducing Cu^2+^ into the electrolyte (Figure 4a), which maintained the high activity of the TiO_2_ photoanode (Figure 1b). However, there was no obvious change in the binding energy of those Ti elements (Figure S10a, Supporting Information), indicating that the surface structure of the TiO_2_ photoanode was not changed after the reaction in the Cu^2+^‐free electrolyte, although it presented a significantly decreased C─C coupling activity. The structures of photoanodes were further explored by Raman spectroscopy as shown in Figure S11 (Supporting Information), where the peaks at 443 and 610 cm^−1^ were attributed to the E_g_ and A_1_ _g_ of rutile TiO_2_, respectively.^[^ ^31^ ^]^ The Raman vibration modes of A_1_ _g_ and E_g_ of TiO_2_ after the reaction exhibited obvious changes. To gain insight into this change, the area ratios of A_1_ _g_ and E_g_ ($S_{A_{1g}}/S_{E_{g}}$) were calculated (Table S3, Supporting Information). We found that the $S_{A_{1g}}/S_{E_{g}}$ of the TiO_2_ reacting in the Cu^2+^‐bearing solution was almost the same as that of the fresh TiO_2_ photoanode, while the $S_{A_{1g}}/S_{E_{g}}$ strength ratio of the TiO_2_ reacting in the Cu^2+^‐free solution was significantly increased.^[^ ^31^ ^]^ The increased $S_{A_{1g}}/S_{E_{g}}$ area ratio implied that the up‐and‐down vibration modes in the TiO_2_ unit cell was inhibited. The constrained up‐and‐down vibrational mode influences the Ti─O bond in the TiO_6_ octahedron to converge, and affects the stretching of the shorter four Ti─O bonds. This result again indicated the strong interaction between TiO_2_ and sp^2^‐C.

The intensity‐modulated photocurrent spectroscopy (IMPS) (Figure 4b) was used to dissect the difference in the interfacial charge transport efficiency (η_ct_) for TiO_2_ photoanodes that have been used in the Cu^2+^‐bearing or Cu^2+^‐free solution.^[^ ^32^, ^33^ ^]^ First, the fresh TiO_2_ was utilized to assess the charge transport efficiency in Cu^2+^‐free or Cu^2+^‐bearing electrolyte (Figure S12a, Supporting Information), while the presence of Cu^2+^ did not exert any influence on η_ct_. For used TiO_2_ photoanodes as shown in Figure 4b, the η_ct_ of the TiO_2_ after reaction in Cu^2+^‐bearing solution was 71%, while the η_ct_ of the TiO_2_ after reaction in Cu^2+^‐free solution was only 47.9% (Table S3 and Figure S12b, Supporting Information). The significant difference indicated that the charge‐transfer kinetics of TiO_2_ without Cu^2+^ was much lower than that with Cu^2+^, which can be attributed to the deposition of organics onto the photoanode surfaces in the Cu^2+^‐free electrolyte. It can be seen from the above characterization that the introduction of Cu^2+^ can prevent the surface passivation of TiO_2_ photoanodes (Figure 4c).

To further rule out the possibility that the Cu^2+^ would be deposited onto the TiO_2_ surfaces during the reaction, cyclic voltammetry (CV) tests were conducted on the fresh TiO_2_ (Figure S13a, Supporting Information), the TiO_2_ after the reaction Cu^2+^‐bearing solutions, and the Cu‐modified TiO_2_ (Cu/TiO_2_) (Figure S13b, Supporting Information).^[^ ^32^ ^]^ As shown in Figure 4d, compared with that of the fresh TiO_2_, there were minimal changes after the reaction in Cu^2+^‐bearing solutions. On the contrary, the significant redox peaks associated with Cu oxide reduction (cathodic sweep) could be observed on the Cu/TiO_2_. These results demonstrated that Cu^2+^ is not likely to deposit on the TiO_2_ surface during the C─C coupling reaction in the Cu^2+^‐bearing solution. It was further supported by XPS measurements as shown in Figure 4e and Figure S10d (Supporting Information). The characteristic peaks of the Cu element could be observed on the Cu/TiO_2_, while the signal of Cu on the TiO_2_ surface after reaction in Cu^2+^‐bearing solutions was not detected. Cu/TiO_2_ was used in standard reaction conditions to test performance. After the reaction (Figure S13c, Supporting Information), the characteristic peaks of the Cu element disappeared in XPS results (Figure 4e; Figure S13d–e, Supporting Information), and Cu oxide reduction (cathodic sweep) could not be observed in the CV test (Figure S13f, Supporting Information). Therefore, compared with the utilization of Cu/TiO_2_, the synergistic heterogeneous/homogeneous PEC strategy is more convenient and exhibits greater stability.

**Figure 5:**
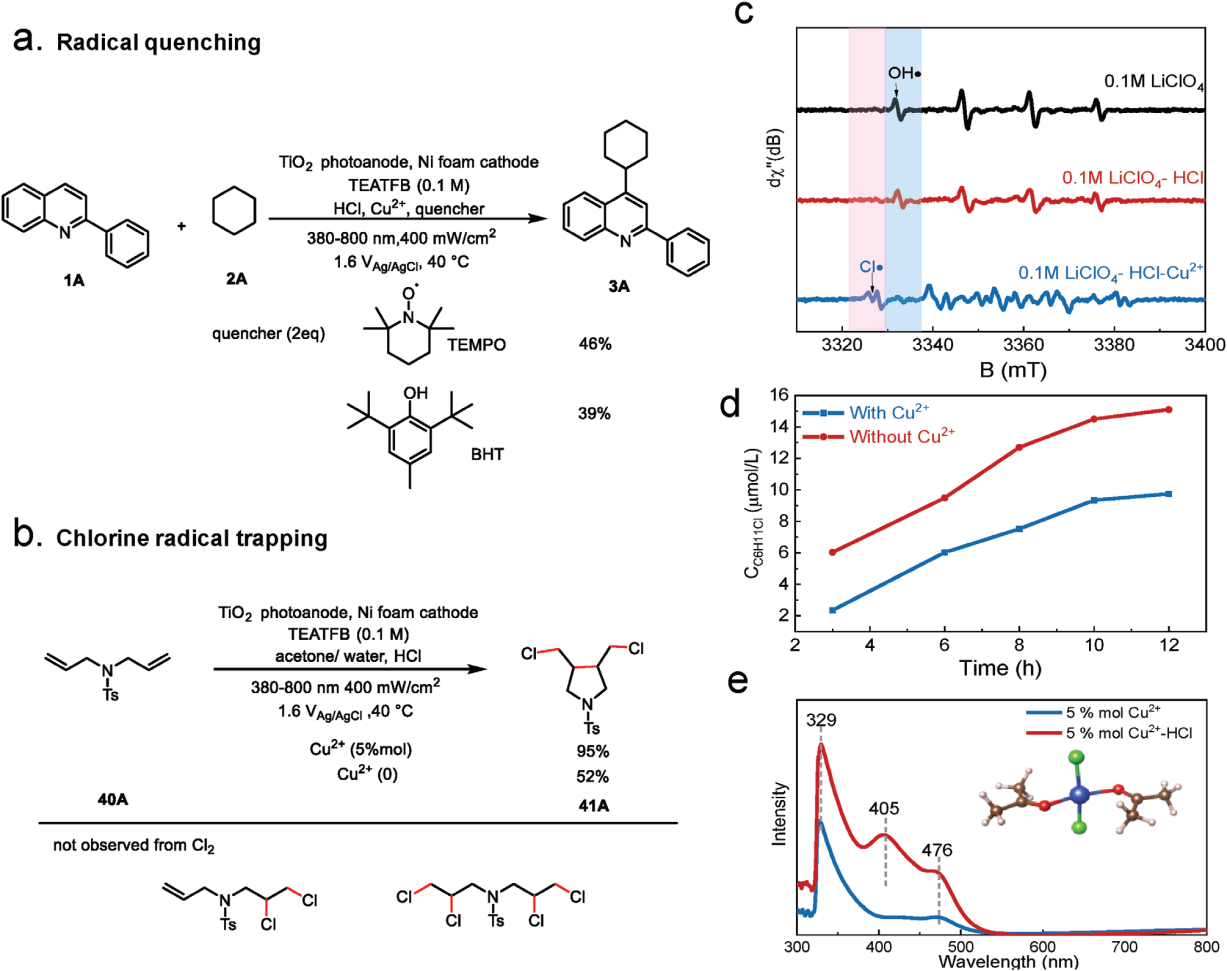
Detection of radicals or intermediates. a) Radical quenching experiments. b) Chorine radical trapping experiments. c) EPR spectra detected from the photoelectrolysis of TiO_2_ under 380–800 nm (400 mW cm^−2^) illumination in acetonitrile and water solution containing DMPO as the spin‐trapping agent. d) Concentration of chlorocyclohexane in standard reaction conditions with Cu^2+^ and without Cu^2+^. e) UV–vis spectra of CuCl_2_ in different conditions and possible structures of CuCl_x_ complex. All were dissolved in a mixture of acetone and water (acetone/water (V/V) = 19:1).

The radicals or intermediates during the C─C coupling reaction were detected to further gain insight into the role of Cu^2+^. It showed that both radical quenchers 2,2,6,6‐tetramethylpiperidine 1‐oxyl (TEMPO) and 3,5‐di‐tert‐4‐butylhydroxytoluene (BHT) exhibited significant inhibition on the formation of **3A** (**Figure** **5**a), confirming the radical characteristic of the PEC process.^[^ ^34^, ^35^ ^]^ The existence of alkyl radicals was further confirmed through the identification of a radical adduct **39A** by using gas chromatography‐mass spectrometry (GC‐MS) (Figure S14, Supporting Information). To further probe the presence of chlorine species, a diallyl sulfonamide **40A** was used under the optimized reaction condition with Cu^2+^ (Figure 5b). A cyclochlorinated compound **41A** was proved by ^1^H NMR and ^13^C NMR. From these results, we can draw a conclusion that the C─C coupling reaction was triggered by the PEC generated Cl**^·^**, while chlorine (Cl_2_) is not active species in our system due to the absence of any specific alkenyl dichloride products.^[^ ^34^, ^35^, ^36^ ^]^

The EPR spectra were further recorded in acetonitrile and water mixtures by using 5,5‐dimethyl‐1‐pyrroline N‐oxide (DMPO) as trapping agent (Figure 5c). In the 0.1 m LiClO_4_, only OH**^·^** was detected. Upon the addition of HCl, the intensity of OH**^·^** signals decreased, but no significant Cl**^·^** signals were observed. This may be due to the low concentration of Cl**^·^** under this condition. Notably, new signals associated with Cl**^·^** were significantly enhanced, and the signals of OH**^·^** almost disappeared after adding Cu^2+^ into the electrolyte.^[^ ^19^, ^28^, ^37^ ^]^ In addition, we used TEMPO as trapping agent to capture Cl**^·^**. When the mixture was subjected to high‐resolution mass spectrometry (HRMS) test, the TEMPO‐Cl was detected, which indicated the formation of Cl**^·^** (Figure S15, Supporting Information). These results confirmed the effective regulation of Cl**^·^** generation during PEC C─C coupling process with the assistance of Cu^2+^. Cl_2_ was generated by dimerization of two Cl**^·^**, which can react with **2A** to produce chlorocyclohexane. We further screen the possibility of Cl_2_ generation. As determined by gas chromatography (GC), the presence of Cu^2+^ significant suppressed the yields of chlorocyclohexane (Figure 5d), indicating that the presence of Cu^2+^ would preferably inhibit the formation of Cl_2_. Furthermore, we purposely replaced CuCl_2_ with CuCl for this reaction. Compared with Cu^2+^, the presence of Cu^+^ exhibited similar product yield and selectivity (Figure S16, Supporting Information). Therefore, Cu^+^ does not affect our proposed mechanism. We further explored the effect of Cu^2+^ on water oxidation reaction (WOR). The oxygen gas was detected by gas chromatography to detect the competition from WOR. The Faraday efficiency of oxygen evolution was relatively low (< 10%) regardless of whether there was Cu^2+^ in the solution (Figure S17, Supporting Information).

The structure of Cu^2+^‐complex was tested by the UV–vis spectroscopy. Figure 5e shows that the absorption peaks of the Cu^2+^‐complex in the acetone were located at 476 and 329 nm. The peak at 329 nm is originated from the n → *π* transition, while the peak at 476 nm is due to the *π* → *π* ^*^ transition.^[^ ^38^ ^]^ The addition of HCl resulted in a new peak at 405 nm and a noticeable color change in the electrolyte from colorless to yellow (Figure S18, Supporting Information), which is consistent with the literature.^[^ ^39^, ^40^ ^]^ Based on UV–vis spectroscopy and previous studies of copper‐chloro complexes in acetonitrile,^[^ ^41^ ^]^ we presented a possible structure (inset of Figure 5e) of the Cu^2+^‐complex, in which two Cl^−^ and two acetone molecules are coordinated to the Cu^2+^ center, i.e., [CuCl_2_(C_3_H_6_O)_2_].

**Figure 6:**
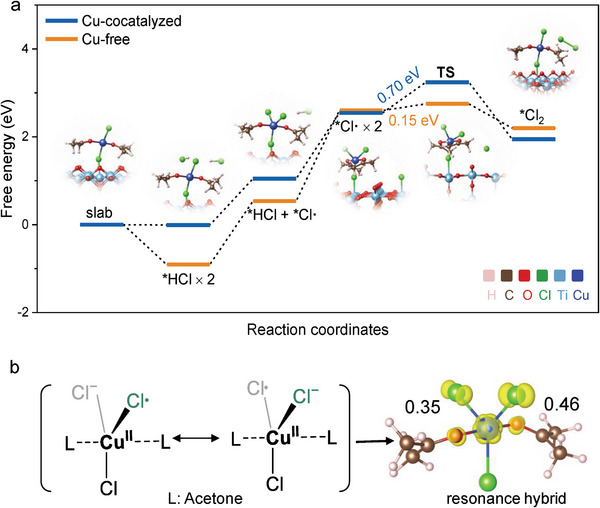
DFT calculations. a) Potential energy surfaces of the Cl^−^ oxidation at the Cu^2+^‐complex/TiO_2_ interface and on the surface of bare TiO_2_. b) the Cl**^·^** resonance structure in the Cu^2+^ solution. The isosurface in yellow corresponds to the spin‐charge density, and the numerical values of 0.35 and 0.46 represent the spin population of those two Cl^−/^ **^·^** species.

We further investigated the production of the Cl**^·^**/Cl_2_ at the Cu^2+^‐complex/TiO_2_ interface or on the surface of bare TiO_2_ by using density functional theory (DFT) calculations (**Figure** **6**). For the Cl^−^ oxidation co‐catalyzed by the Cu^2+^ and TiO_2_, the whole pathway started from the structure of the adsorbed Cu^2+^‐complex on the (110) facet of the TiO_2_ photoanode as depicted in Figure 6a (slab). The adsorption of the Cu^2+^‐complex is a spontaneous process with a large adsorption energy of −1.58 eV, which positions the Cu^2+^‐complex within the Helmholtz layer of the TiO_2_ photoanode. Besides, the valence state of Cu sites in the complex almost remains +2 within the catalytic cycle via Bader charge analysis as revealed in Figure S19 (Supporting Information). For the Cl^−^ oxidation process, two HCl molecules were introduced as the chlorine source to elucidate the energy difference in the formation of Cl_2_ in these two pathways (Figure 6a). The first Cl**^·^** was produced by transferring a proton and electron from one HCl molecule, forming a five‐coordinated CuCl_3_(C_3_H_6_O)_2_ intermediate (Figure 6b). This process exhibits a lower formation energy of 1.06 eV compared with that of 1.45 eV on the bare TiO_2_. Notably, as shown in Figure 6b, there were two resonance structures for the CuCl_3_(C_3_H_6_O)_2_ intermediate, displaying the de‐localization of spin electrons. These two Cl atoms coordinated to the centered Cu^2+^ site present a spin population of 0.35 and 0.46, indicating of a more stable Cl**^·^**. As a result, it required a high energy barrier of 0.70 eV for the formation of Cl_2_ at the Cu^2+^‐complex/TiO_2_ interface, compared with that of 0.15 eV on the surface of bare TiO_2_. These results indicate that the Cu^2+^‐complex/TiO_2_ interface facilitates Cl**^·^** formation while inhibiting Cl_2_ production, thereby promoting the HAT efficiency.

**Figure 7:**
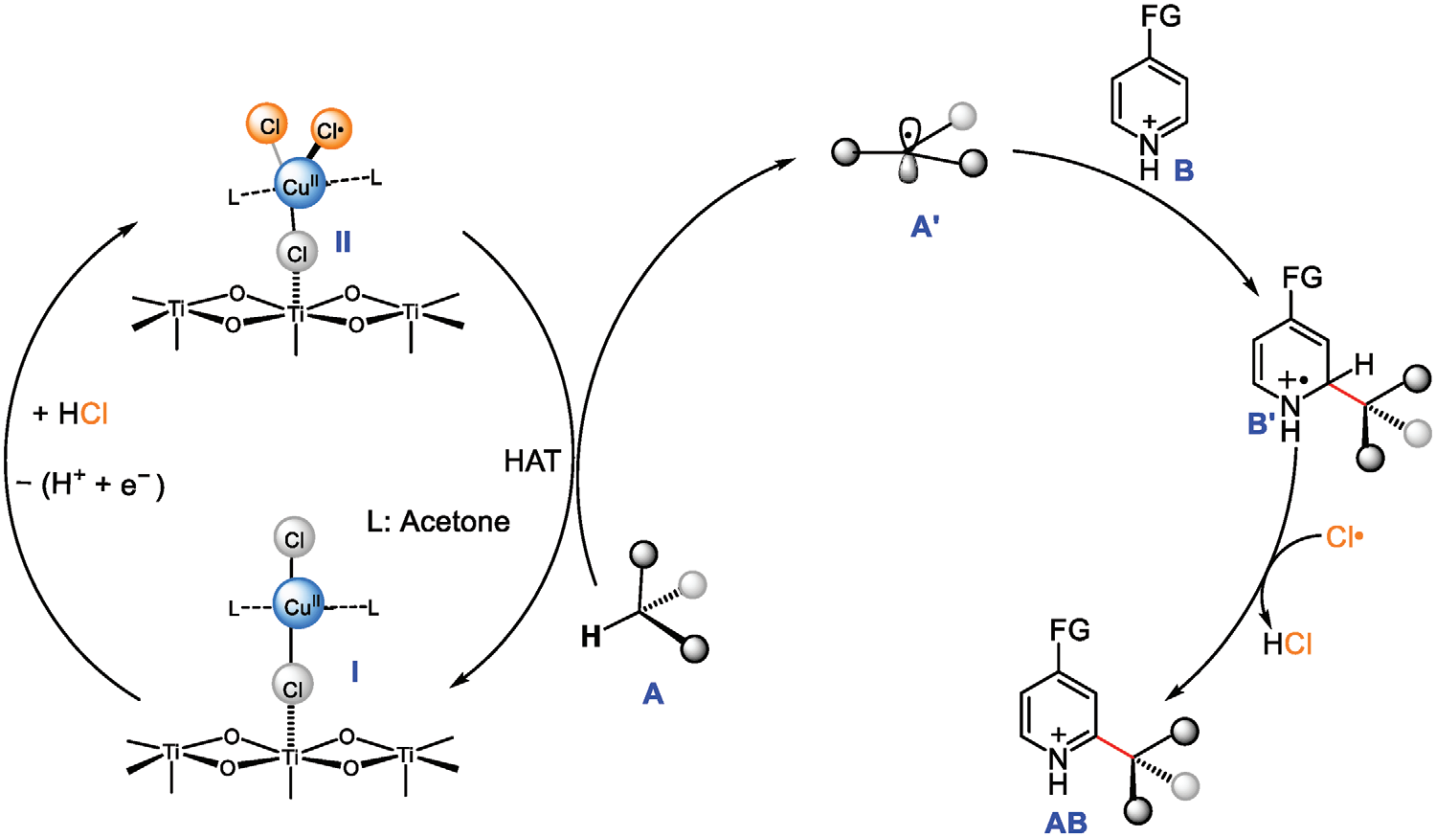
The proposed mechanism for the Cu^2+^ co‐catalyzed Minisci radical coupling reaction. Black and gray ball represent hydrogen atoms.

The proposed mechanism of C─C coupling reaction via the PEC strategy by using TiO_2_ is illustrated in **Figure** **7**. First, CuCl_2_ in the system is coordinated with acetone to form CuCl_x_ complex (**I**). Subsequently, the **I** is adsorbed on the TiO_2_ surface through electrostatic interactions. Then, the HCl adsorbed near the Cu site is oxidized by photogenerated holes to form pentacordinate CuCl_x_ complex (**II**). The Cl**^·^** acts as a highly efficient HAT agent, reacting with cyclohexane to generate alkane radical (**A′**) and release the **I** for the next cycle spontaneously. Then **A′** combines with the protonated aromatics (**B**) to get the intermediate of the radical cation (**B′**), and Cl**^·^** plays a crucial role to rearomatize the intermediate and yields the final product (**AB**). In addition, the **A′** readily undergoes a reaction with Cl_2_ to yield chlorocyclohexane as a byproduct. The presence of Cu^2+^ can inhibit the interaction of Cl**^·^** to form Cl_2_ and reduces unwanted formation of alkyl chloride.

The proposed structure of CuCl_x_ complex may provide insights into the underlying mechanisms responsible for the high regioselectivity of the presented C─C coupling method. First, pentadentate CuCl_x_ complex (Figure S20, Supporting Information; **II**) intermediates might be engaged in hydrogen bonding interactions with a suitable radical nucleophile as well as its quinoline counterion (Figure S20, Supporting Information; **III**). The subsequent step involves elimination of HCl from intermediate **III** to obtain intermediate **IV**. The hydrogen bonding would favor the alkylation at the C2 position. After radical addition, the CuCl_x_ complex transitions from intermediate **IV** to the next cycle, while simultaneously acquiring intermediate **V**. Finally, Cl**^·^** would function as the single‐electron oxidant required to rearomatize the heteroarene. This hypothesis is consistent with the result that CuCl_x_ complex leads to a high regioselectivity.

For the continuous production, we sought to construct a flow electrolyzer. TiO_2_ was used as the anode (2 cm × 2 cm) and Ni foam as the cathode (2 cm × 2 cm), and the electrolytic cell was connected to a 500 mL glass bottle serving as a reservoir for the reaction mixture (Figure S21, Supporting Information). As illustrated in Figure S22 (Supporting Information), the TiO_2_ exhibited a significantly enhanced photocurrent under 1 W cm^−2^ illumination, reaching a maximum of 10 mA at 1.6 V_Ag/AgCl_. The current can maintain stability under these conditions for the reaction. The synthesis of **3A** could be conducted on a gram scale with production rate of 105.2 mmol cm^−2^ h^−1^ by employing this flow setup.

## 3 Conclusion

In summary, we developed a synergistic heterogeneous/homogeneous PEC strategy to achieve a controllable radical‐induced C─C coupling reaction mediated by Cu^2+^ on TiO_2_ photoanodes, which can be performed in the absence of exogenous oxidants. The presence of Cu^2+^ at the TiO_2_/electrolyte interfaces facilitates the reaction between 2‐phenylquinoline and cyclohexane, resulting in a maximum yield of 90% with a selectivity of 95% and long‐term stability over 100h. This method can also enable aliphatic compounds with a variety of inert C(sp^3^)─H configurations to be coupled with a wide range of aromatics. The presence of Cu^2+^ promotes the formation of Cl**^·^** and restrains the formation of Cl_2_. The formation of the CuCl_x_ complex plays an important role in the high regioselectivity. We believe that the present study would provide insights for future PEC organic transformations.

## Conflict of Interest

The authors declare no conflict of interest.

## Author Contributions

Q.L. and K.D. contributed equally to this work. Y.Z. directed the project. Q.L. carried out most experiments. Q.L, Y.Z., and K.D. wrote the manuscript, with input from others. K.D. conducted computational studies. S.L. and L.W helped with the IMPS experiments and results simulation. All the authors analyzed the results and reviewed the paper.

## Supporting information



Supporting Information

## Data Availability

The data that support the findings of this study are available from the corresponding author upon reasonable request.
