# Supplementary material for: Regulating Chlorine and Hydrogen Atom Transfer for Selective Photoelectrochemical C─C Coupling by Cu‐coordination Effect at Semiconductor/Electrolyte Interfaces
Source: Adv Sci (Weinh). 2024 Oct 24;11(46):2408767. doi: 10.1002/advs.202408767 (PMC11633461; doi:10.1002/advs.202408767)
Supplement: Supplementary file 1 — Supporting Information [file ADVS-11-2408767-s001.docx]

**Regulating Chlorine and Hydrogen Atom Transfer for Selective Photoelectrochemical C–C Coupling by Cu-coordination Effect at Semiconductor/Electrolyte Interfaces**

Qiaozhen Li,^1,2#^ Kun Dang,^1,2#^ Lei Wu,^1,2^ Siqin Liu,^1,2^ Yuchao Zhang,^1,2^* and Jincai Zhao^1,2^

1 Key Laboratory of Photochemistry, CAS Research/Education Center for Excellence in Molecular Sciences, Institute of Chemistry, Chinese Academy of Sciences, Beijing 100190 (P. R. China)

2 University of Chinese Academy of Sciences, Beijing 100049 (P. R. China)

E-mail: [yczhang@iccas.ac.cn](mailto:yczhang@iccas.ac.cn)

# These authors contributed equally.

1. **Materials.**

Titanium butoxide was purchased from Alfa Aesar. The fluorine-doped tin oxide substrates (FTO, 2.2 mm) were purchased from Nippon Plate Glass Co., Ltd. Other reagents were purchased from Alfa Aesar, Acros, Innochem, Aladdin, and J&K Scientific companies and were used without further purification unless otherwise specified.

1. **Preparation of photoanodes.**
   1. **TiO_2_ photoanodes**.

TiO_2_ photoanodes were prepared on fluorine-doped tin oxide substrates (FTO) through hydrothermal regrowth method.^1^ Generally, FTO substrates (1 × 2 cm^2^) were first sonicated with acetone, ethanol and water for 30 min each and dried. Then, 1 mL of titanium butoxide was added into H_2_O/HCl mix solution with equal volume (25 mL) of H_2_O and HCl (36.5–38 wt%) under continuous stirring. The mixture should be stirred at ambient temperature for half an hour. 7.2 mL resulting solution and the pretreated FTO substrate were transferred and sealed in a Teflon-lined stainless-steel autoclave (20 mL) and heated to 150 °C for 10h. The film was removed from the autoclave, washed thoroughly with distilled water and dried in the air. The film was ultimately subjected to annealing at 450 °C in an ambient environment for 1.5h (heating rate: 10 °C min^−1^) to obtain the TiO_2_ photoanode.

- 1. **Cu-modified** **TiO_2_ photoanodes**

A bare TiO_2_ photoanode was used as WE, and an electrodeposition was conducted in the 0.3 M NaClO_4_ aqueous solution containing 1 mM CuSO_4_.^2^ The deposition potential was optimized at −1.0 V_Ag/AgCl_, while the optimal duration for deposition was determined to be 20s. The resulting Cu-modified TiO_2_ photoanodes were rinsed using DI water and dried with N_2_ gas.

**3. Photoanodes characterization.**

The surface morphology of samples was characterized by scanning electron microscope (SEM, Hitachi, S4800) at a working voltage of 10 kV. X-ray diffraction (XRD) patterns of TiO_2_ and FTO blank were collected on an X-ray diffraction instrument (Rigaku, D/max 2500) with 5° min^-1^ over a 2θ value range from 20° to 80°. Ultraviolet-visible diffuse (UV-vis DRS) reflectance spectrum measurements were performed using a Hitachi U-3900 spectrometer spectrophotometer with BaSO_4_ as the reference standard. X-ray photoelectron spectroscopy (XPS) was conducted on the Thermo Scientific ESCALab 250Xi using 200 W monochromatic Al Kα radiation. The hydrocarbon C1s line at 284.8 eV from adventitious carbon was used for calibration. Raman spectroscopy provides more material structure information (LabRAM Odyssey, HORIBA).

1. **PEC measurements.**

PEC measurements were conducted in a three-electrode electrochemical cell under the control by an electrochemical workstation (CHI760E for photoelectrolysis experiments, Autolab PGSTAT302N for PEC characterizations), unless otherwise specified. The photoelectrochemical oxidation was performed in an undivided cell, TiO_2_ was used as a working electrode with Ag/AgCl reference electrode and a Ni foam counter electrode. A Xenon lamp (CEAULIGHT, CEL-HXF300-T3) with an AM 1.5G and 380–800 nm filter was used as the light source. Its irradiation intensity was determined as 400 mW cm^-2^ by a radiometer (CEAULIGHT, CEL-NP2000-2A). Linear sweep voltammetry (LSV) curves were conducted at a scan rate of 50 mV s^-1^ if not separately labeled. Before photoelectrolysis experiments, the electrolyte was bubbled with Ar gas flow for 15 min to remove air. Photoelectrolysis was conducted through chronoamperometry at an applied potential for 13h. For intensity-modulated photocurrent spectroscopy (IMPS) measurements,^2-3^ a DC light intensity of 70 mW cm^-2^ with a modulated intensity of 10% was employed, while the frequency range varied from 10 kHz to 0.1 Hz with 20 points per decade. IMPS spectra were analyzed using Nova 2.1.4 software. The light source for PEC characterizations was a 470 nm LED (Metrohm, LDC470).

1. **Product analysis.**

^1^H NMR, ^13^C NMR spectra were recorded on a Bruker AV400 (FT, 400 MHz for 1H; 100 MHz for 13C). The abbreviations used for explaining the multiplicities were as follows: s = singlet, d = doublet, t = triplet, q =quartet, m = multiplet, br = broad. The GC-MS measurements were run on an Agilent GC-MS instrument with a HP-5ms GC column and electron ionization ion source. The solvent delay time was 3 min.

1. **Computational details**

The spin-polarized DFT calculations were carried out using the PBE functional and DZVP basis sets in the CP2K package,^4-5^ and the dispersion correction was applied in all calculations with the Grimme D3 method.^6^ Only Γ point was used in all calculations here due to the large size of the supercell. The cutoff of 500 Ry and GTH pseudopotential were used for geometry optimization and energy calculations. The energy convergence for the self-consistent field calculation was set to 10^−6^ Hartree, and the largest forces on atoms were set to less than 4.5×10^−4^ Hartree/Bohr. The cell of the rutile TiO_2_ (4.653 Å × 4.653 Å × 2.969 Å) was optimized based on a 3×3×4 supercell model, and the optimized cell vectors are 4.619 Å × 4.619 Å × 2.965 Å. Then the TiO_2_ (110) facet was constructed for the catalytic calculations according to our previously reported experimental data^1^, exhibiting a 4-layer 4 × 2 × 1 supercell with a 15 Å vacuum layer. The structure of the Cu^2+^-complex was fabricated based on the previous work^7^ and our experimental results, and the final configuration was determined due to its lowest total energy among others. The free energy correction was conducted using Shermo2.4 software developed by Lu et. al..^8^ The transition state (TS) was located with the climbing image nudged elastic band (CI-NEB) method integrated into CP2K.

The adsorption energy was calculated via the equation below:

$E_{ad}=E_{slab+mol}-E_{slab}-E_{mol}$ (1)

1. **Procedures for the photoelectrolysis**

**7.1 Representative procedure for the synthesis of 3A:**

In a 10 mL undivided cell equipped with a magnetic stirring bar, heteroarene **1A** (0.1 mmol), CuCl_2_ (0.005 mmol), acetone (4.75 mL), H_2_O (0.25 mL), HCl (100 μL) and alkane **2A** (0.3 mL) were added. Before photoelectrolysis experiments, the electrolyte was bubbled with Ar gas flow for 15 min to remove air. A Xenon lamp with an AM 1.5G and 380–800 nm filter was used as the light source. The photoelectrolysis was performed via chronoamperometry under an applied potential for 13h under 40 °C (Water bath). The reaction was quenched with saturated NaHCO_3_ and extracted with ethyl acetate (3 × 5 mL). The combined organic layer was dried over anhydrous Na_2_SO_4_, filtered and concentrated under reduced pressure. The compound **3A** was isolated by preparative thin-layer chromatography (90% yield).

**7.2 Procedure for Gram-Scale Reaction:**

In a 50 mL undivided cell equipped with a magnetic stirring bar, heteroarene **1A** (1 g, 4.88 mmol), CuCl_2_ (5% mmol), acetone (19 mL), H_2_O (1 mL), HCl (500 μL) and alkane **2H** (4.5 mL) were added. Before photoelectrolysis experiments, the electrolyte was bubbled with Ar gas flow for 15 min to remove air. A Xenon lamp with an AM 1.5G and 380–800 nm filter was used as the light source. The photoelectrolysis was performed via chronoamperometry under an applied potential for 13h under 40 °C (Water bath). The reaction was quenched with saturated NaHCO_3_ and extracted with ethyl acetate (3 × 25 mL). The combined organic layer was dried over anhydrous Na_2_SO_4_, filtered and concentrated under reduced pressure. The residue was chromatographed through silica gel eluting with ethyl acetate/hexanes giving compound **10A** (0.215 g).

**8. Mechanistic studies**

**8.1 Radical quenching experiment**

In a 10 mL undivided cell equipped with a magnetic stirring bar, heteroarene **1A** (0.1 mmol), CuCl_2_ (0.005 mmol), acetone (4.75 mL), H_2_O (0.25 mL), HCl (100 μL) and radical quencher (TEMPO or BHT, 0.2 mmol) were added. The mixture was then added alkane **2A** (0.3 mL). Before photoelectrolysis experiments, the electrolyte was bubbled with Ar gas flow for 15 min to remove air. A Xenon lamp with an AM 1.5G and 380–800 nm filter was used as the light source. The photoelectrolysis was conducted through chronoamperometry at an applied potential for 13h under 40 °C (Water bath). The reaction was quenched with saturated NaHCO_3_ and extracted with ethyl acetate (3 × 5 mL). The combined organic layer was dried over anhydrous Na_2_SO_4_. The volatiles were removed under reduced pressure to obtain the crude product. The formation of **3A** was significantly suppressed by radical quenchers (46% with TEMPO, 39% with BHT), which suggested the reaction proceeds through a radical-involved pathway. When TEMPO was use as the radical quencher, the by-product **39A** was detected by GC-MS (Fig. S14), which could be evidence for the cyclohexyl radical formation.^9^

**8.2 Chlorine radical trapping experiment**

In a 10 mL undivided cell equipped with a magnetic stirring bar, chlorine radical acceptor **40A** (25.1 mg, 0.1 mmol), CuCl_2_ (0.005 mmol), acetone (4.75 mL), H_2_O (0.25 mL) and HCl (100 μL) were added. Before photoelectrolysis experiments, the electrolyte was bubbled with Ar gas flow for 15 min to remove air. A Xenon lamp with an AM 1.5G and 380–800 nm filter was used as the light source. The photoelectrolysis was performed via chronoamperometry under an applied potential for 13h under 40 °C. (Water bath). The reaction was quenched with saturated NaHCO_3_ and extracted with ethyl acetate (3 × 5 mL). The combined organic layer was dried over anhydrous Na_2_SO_4_. The volatiles were removed under reduced pressure to obtain the crude product. The isolated **41A** was obtained in 95% yield by preparative thin-layer chromatography using Hex/EtOAc (10:1) as the eluent. When there was no Cu^2+^ in the system, the isolated **41A** was obtained in 52% yield by preparative thin-layer chromatography. The formation of **41A** in this reaction suggested the reaction might proceed with the formation of chlorine radical, as no dichlorination of alkene was detected by ^1^H NMR.^10-11^

**8.3 Effect of copper on by-products**

In a 10 mL undivided cell equipped with a magnetic stirring bar, heteroarene **1A** (0.1 mmol), CuCl_2_ (0.005 mmol), acetone (4.75 mL), H_2_O (0.25 mL) and HCl (100 μL) were added. The mixture was then added alkane **2A** (0.3 mL). Before photoelectrolysis experiments, the electrolyte was bubbled with Ar gas flow for 15 min to remove air. A Xenon lamp with an AM 1.5G and 380–800 nm filter was used as the light source. Every few hours, 100 μL of electrolyte solution was collected from the photoelectrochemical cell and diluted it with 1 mL of acetone, the mixed solution was analyzed by GC. Calibration curves of chlorocyclohexane were obtained by GC to quantify the amount of chlorocyclohexane formed.

**8.4. Electron paramagnetic resonance (EPR) measurements**

5,5-Dimethyl-1-pyrroline N-oxide (DMPO) was used as the spin trapping agent.^12-14^ In general, 100 μL 0.2 M DMPO solution was added to the electrolyte through a pipette during the photoelectrolysis. It was necessary to ensure that the pipette and photoanodes were in close contact, they would in-situ react with the DMPO to form corresponding species. Subsequently, the final solution was collected for the EPR measurements. Finally, capillary tubes containing the above collected solutions were used for EPR measurements. The parameters were as follows: Bruker E500, 3350 G center of field; 9.412911 GHz frequency; 1G modulation amplitude; 40.96 s concersion time; 40.96 s time constant; 5.098 mW microwave power; 100 G scanwidth; 41.94 s sweep time.

1. **Flow electrolyzer**

The decagram scale synthesis of **3A** was conducted in a 50 mL undivided cell with TiO_2_ (2 cm × 2 cm) as the anode, a Ni foam cathode (2 cm × 2 cm). A 500 mL glass bottle as a reservoir for reaction mixture was linked with the electrolytic cell. The continuous circulation of solution between the cell and the reservoir was achieved by a peristaltic pump through Teflon catheter with a flow rate 40 mL min^−1^. In a 50 mL undivided cell equipped with a magnetic stirring bar, heteroarene **1A** (80 mg, 0.4 mmol), CuCl_2_ (5% mmol), acetone (19 mL), H_2_O (1 mL), HCl (400 μL) and alkane **2A** (1.2 mL) were added. The solution in 500 mL glass bottle consisted **1A** (5 g, 9.7 mmol), TEATFB (10.8 g), CuCl_2_ (5% mmol), acetone (427.5 mL), H_2_O (22.5 mL), HCl (10 mL) and alkane 2A (30 mL). Before photoelectrolysis experiments, the electrolyte was bubbled with Ar gas flow for 15 min to remove air. A Xenon lamp with 300–800 nm filter (1 W/cm^2^) was used as the light source. The photoelectrolysis was conducted via chronoamperometry at an applied potential under 40 °C (Water bath).

Fig. S1. Linear sweep voltammetry (LSV) curves of a TiO_2_ photoanode. The measurements were carried out under the illumination of 380–800 nm (400 mW/cm^2^).


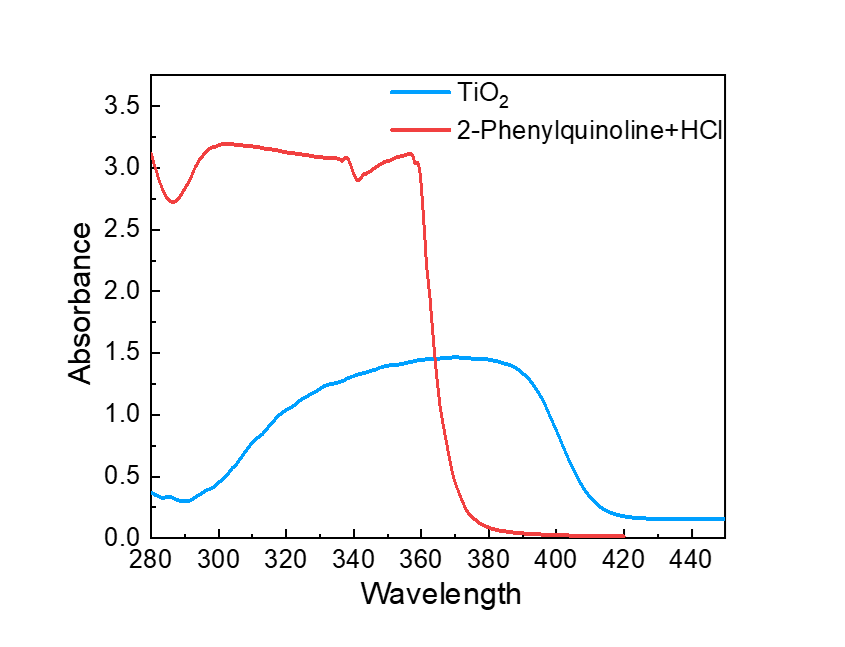


Fig. S2. UV-vis DRS of TiO_2_ and the UV-vis of 2-Phenylquinoline with HCl liquid.

Fig. S3. Reaction setup for the PEC reaction. (**a**) Reactions were carried out in a three-electrode cell. TiO_2_ as the photoanode, Ni foam as the cathode. (**b**) Photographs of the undivided cell for PEC C–C coupling.

Fig. S4. TiO_2_ photoanodes after reaction in (**a**) Cu^2+^-free solution and (**b**) Cu^2+^-bearing solution.

Fig. S5. (**a**) LSV curves of the TiO_2_ photoanode under 380–800 nm illumination and in the dark with or without Cu^2+^. (**b**) Chronoamperometry for the photoelectrolysis process at a fixed potential of 1.6 V_Ag/AgCl_.

Fig. S6. Comparison of performance under different light intensities. (**a**) Linear sweep voltammetry (LSV) curves of the TiO_2_ photoanode. (**b**) Selectivity and yield of reaction products.

The high light intensity was utilized to enhance the current density, thereby attaining a greater yield within a fixed time. As shown in Fig. S6a, the increased light intensity led to a higher current density. We further conducted photoelectrolysis experiments to investigate the influence of the light intensity on the yield and selectivity (Fig. S6b). With other conditions unchanged, the yield of the **3A** obtained under 100 mW/cm^2^ was only 57%, which was much lower than that of 90% under 400 mW/cm^2^. The selectivity of the product remained 95% irrespective of variations in light intensity.

Fig. S7. Roles of Cu^2+^ in product regioselectivity. (**a**) ^1^H NMR of **19A**. (**b**) ^1^H NMR of **19B**. Quinoline reacts with cyclohexane in (**c**) Cu^2+^-bearing solution and (**d**) Cu^2+^-free solution. # represents the characteristic peak of C2-position alkylated products. _*_ represents the characteristic peak of C4-position alkylated products.

Fig. S8. SEM of TiO_2_. (**a**) Fresh TiO_2_. (**b**) TiO_2_ after reaction with Cu^2+^. (**c**) TiO_2_ after reaction without Cu^2+^.

Fig. S9. X-ray diffraction patterns of the fresh TiO_2_, the TiO_2_ after reaction with Cu^2+^ and the TiO_2_ after reaction without Cu^2+^. a.u.: arbitrary units.

Fig. S10. XPS measurements of TiO_2_. (**a**) Ti 2p, (**b**) Cl 2p, (**c**) O 1s and (**d**) Cu 2p XPS spectra of the fresh TiO_2_, the TiO_2_ after reaction with Cu^2+^ and the TiO_2_ after reaction without Cu^2+^.

The high-resolution XPS (HR-XPS) results of Ti 2p for various TiO_2_ photoanodes were exhibited in Fig. S10a, where the peaks at 464.5 and 458.7 eV were assigned to the 2p_1/2_ and 2p_3/2_ of Ti^4+^, respectively.^15^ As shown in Fig. S10b, the XPS measurement revealed the presence of characteristic Cl 2p XPS peaks at 198.1 eV (Cl 2p_3/2_) and at 199.8 eV (Cl 2p_1/2_) for TiO_2_ after reaction. The O 1s XPS spectra illustrated typical oxygen adsorption peak (Fig. S10c). Notably, the signal of the Cu element was not detected on the TiO_2_ surfaces after the reaction in the Cu^2+^-bearing electrolyte as shown in Fig. S10d. In other words, the Cu atom was not incorporated onto the TiO_2_ surfaces during the Cl**·**-mediated C–C coupling reaction, implying that the Cu^2+^ worked through a homogeneous pathway.

Fig. S11. Raman spectra of the fresh TiO_2_, the TiO_2_ after reaction with Cu^2+^ and the TiO_2_ after reaction without Cu^2+^.

Fig. S12. IMPS measurements. (**a**) Normalized IMPS results of the fresh TiO_2_ in standard reaction conditions with Cu^2+^ and without Cu^2+^. (**b**) The pristine IMPS results of the fresh TiO_2_, the TiO_2_ after reaction without Cu^2+^ and the TiO_2_ after reaction with Cu^2+^_._ All were performed in standard reaction conditions without Cu^2+^.

Fig. S13. Physical characterizations and performance of Cu/TiO_2_. (**a**) SEM of the fresh TiO_2_. (**b**) SEM of the Cu/TiO_2_. (**c**) Reaction performance for the C–C coupling on the Cu/TiO_2_ photoanode in two batches. The reaction was carried out in acetone/water solution with 0.1 M TEATFB and 100 μL HCl at 1.6 V_Ag/AgCl_ under the illumination of 380–800 nm (400 mW cm^−2^) with 13h for every batch. The photoanode was washed by deionized water and acetonitrile after each batch reaction. (**d**) O 1s XPS and (**e**) Ti 2p XPS spectra of the fresh TiO_2_, the Cu/TiO_2_ before and after reaction. (**f**) The CV curves of the Cu/TiO_2_ before and after reaction in 0.3 M NaClO_4_ aqueous solutions. The scan rate was 10 mV·s^-1^.

To rule out the possibility that the surface-modified Cu species loaded on TiO_2_ acted to improve the yield, Cu^2+^ was purposely electrodeposited on TiO_2_ photoanodes (denoted as Cu/TiO_2_). As for the Cu/TiO_2_, a large amount of 10~50 nm Cu nanoparticles were electrodeposited on the TiO_2_ nanorods (Fig. S13b). Subsequently, the Cu/TiO_2_ was used to test the C–C coupling reaction in the Cu^2+^-free electrolyte. The first cycle showed a decent yield of 82%, but the yield of the second cycle was greatly reduced (Fig. S13c), which was the same with the fresh TiO_2_. To further identify the difference of Cu/TiO_2_ composition before and after the reaction, the XPS analysis of the fresh TiO_2_, Cu/TiO_2_ before and after the reaction were conducted. The Cu 2p_3/2_ peak at 932.9 eV and the Cu 2p_1/2_ peak at 952.8 eV can correspond to Cu^0^ or Cu^+^ (**Fig. 4d**).^15^ Compared to the Cu/TiO_2_ before the reaction, the characteristic peaks of Cu element was not be observed on the Cu/TiO_2_ after reaction surface. As shown in Fig. S13f, The CV obtained from the Cu/TiO_2_ after reaction revealed a single peak exclusively associated with the oxygen reduction reaction (ORR).^16^ These results demonstrated that there was no strong adsorption or deposition of Cu^2+^ on the TiO_2_ surface after photoelectrolysis in the Cu^2+^-bearing solution.

Fig. S14. MS spectra of 39A.

Fig. S15. HRMS analysis of chlorine radicals trapping experiment.

We used TEMPO as a trapping agent to capture Cl**·**, which was further analyzed by High-Resolution Mass Spectrometry (HRMS). As shown in Fig. S15, the measured m/z value was 192.1152, and the derived formula corresponded to C_9_H_19_ONCl. This was consistent with our anticipated m/z value and chemical structure, which indicated the formation of Cl**·** in our case.

Fig. S16. Identification of the role of Cu^+^ in C−C coupling reaction. (**a**) Comparison of CV (scan rate 50 mVs^-1^) of the TiO_2_ in 0.1 M TEATFB solutions containing 100 μL HCl and 0.1 M TEATFB solutions containing 100 μL HCl and CuCl. (**b**) Comparison of UV-vis spectra of CuCl_2_ solution, CuCl solution and CuCl solution after electrolysis. All were dissolved in a mixture of acetone and water (Acetone/water (V/V) = 19:1). (**c**) The yields and selectivity of **3A** at 1.6 V_Ag/AgCl_ for the TiO_2_ photoanode in different solutions.

To further determine if Cu^+^ existed and took effect in this reaction system, a series of tests were performed. The cyclic voltammogram (CV) obtained from CuCl displayed an oxidation peak at –0.03 V_RHE_ which was associated with Cu^+^ oxidation, as depicted in Fig. S16a. Obviously, the Cu^+^ oxidation potential is much lower than the valence band position of TiO_2_ (~3.2 V_RHE_), indicating that Cu^+^ is easy to be oxidized to Cu^2+^ during the electrolytic reaction. In addition, the oxidation of Cu^+^ was further verified by UV–vis spectroscopy. As shown in Fig. S16b, the absorption peaks of Cu^2+^ were observed but nearly negligible for that of Cu^+^ within 300−800 nm. However, the absorption peak exhibited a substantial increase after 30 min of electrolysis in the Cu^+^ electrolyte. Therefore, these results indicated that even in the presence of a case where Cu^2+^ is reduced to Cu^+^ under illumination, the resulting Cu^+^ undergoes facile oxidation to form Cu^2+^.

We further replaced CuCl_2_ with CuCl for a catalytic reaction (Fig. S16c). Compared with Cu^2+^, the case in the presence of Cu^+^ exhibited similar product yield and selectivity. This is consistent with our proposed conclusion that Cu^+^ readily undergoes oxidation to Cu^2+^ in this reaction system. The amount of the charge required to convert Cu^+^ to Cu^2+^ was negligible, which was significantly lower than the total charge of the reaction. Based on these experimental data, we believe that Cu^+^ does not affect our proposed mechanism.

Fig. S17. FE of O_2_ at 1.6 V_Ag/AgCl_ for TiO_2_ photoanodes in the 0.1 M TEATFB, 100 μL HCl solution with Cu^2+^ or without Cu^2+^.

The oxygen gas was detected by gas chromatography to detect the competition from the water oxidation reaction (WOR). The Faraday efficiency of oxygen evolution was relatively low (< 10%) regardless of whether there was Cu^2+^ in the solution (Fig. S17).

Fig. S18. Image showing the color of solutions under different conditions. (**a**) Color of aqueous Cu^2+^ (5% mol) solution. (**b**) Acetone shifts the color of aqueous Cu^2+^ (5% mol) solutions from blue to pale yellow. (**c**) Addition of HCl to solution (**b)** results in a yellow color change in the solution.

Fig. S19. Calibrated valence states of Cu site using Bader charge analysis. Note: The dots of “with 1 Cl**·**” and “with 2 Cl**·**” almost overlapped.

The Bader charge analysis based on DFT calculations was conducted to understand the valence of the Cu site in the Cu-complex on the photoanode surface during the reaction. We used four representative materials as the reference samples, i.e., metal Cu (0), Cu_2_O (+1), CuO (+2) and CuOOH (+3), to establish the relationship between the Bader charge and the valence states of the Cu site. The calculated Bader charge as the function of valence states of Cu sites was obtained by linear fitting as shown in Fig. S19. We further calculated the Bader charge of Cu sites in isolated Cu-complexes, slab (Cu-complex adsorbed on the TiO_2_ surface), and the intermediates during the reaction with 1 or 2 Cl**·**. According to the linear relation as revealed in Fig. S19, the valence state of the Cu site in the Cu-complex almost remains +2 during the reaction process, although the Bader charge exhibits a slight difference due to the reorganization of the complex.

Fig. S20. The mechanism for enabling regioselectivity with CuCl_x_ complex.


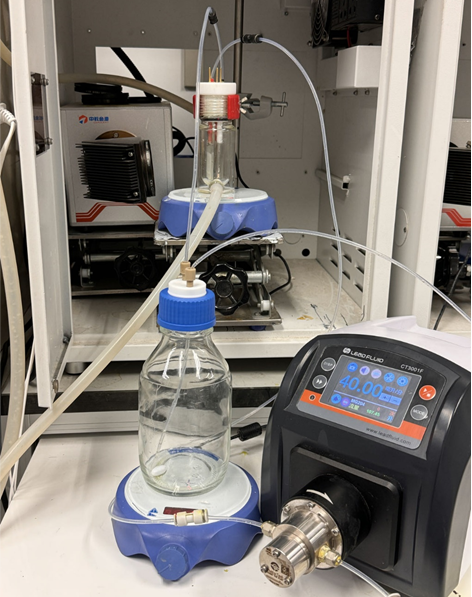


Fig. S21. Flow reactor for the decagram scale reaction. 50 mL undivided cell with TiO_2_ (2 cm × 2 cm) as the anode, Ni foam cathode (2 cm × 2 cm). 500 mL glass bottle as a reservoir for reaction mixture was linked with the electrolytic cell. Photoelectrolysis process at a fixed potential of 1.6 V_Ag/AgCl_ under 300–800 nm illumination (1 W/cm^2^). The flow rate was 40 mL min^–1^.

Fig. S22. (a) LSV curves of the TiO_2_ photoanode under 300–800 nm illumination (1 W/cm^2^). (b) Chronoamperometry for the photoelectrolysis process at a fixed potential of 1.6 V_Ag/AgCl_ under 300–800 nm illumination (1 W/cm^2^).

**Table S1. Additional optimization of reaction conditions^[a]^.**

| Entry | Electrolyte | T (^o^C) | V_Ag/AgCl_ | Conversion (%) | Yield (%) ^[b]^ |
| --- | --- | --- | --- | --- | --- |
| 1 | TEATFB | 40 | 1.6 | 95 | 90 |
| 2 | TEATFB | 40 | 1.2 | 85 | 85 |
| 3 | TEATFB | 40 | 0.8 | 78 | 78 |
| 4 | TEATFB | 25 | 1.6 | 60 | 57 |
| 5 | TEATFB | 50 | 1.6 | 90 | 85 |
| 6 | TBABF4 | 40 | 1.6 | 70 | 65 |
| 7 | TBAPF6 | 40 | 1.6 | 32 | 30 |
| 8 | TMMTFB | 40 | 1.6 | 85 | 83 |
| 9 | LiClO_4_ | 40 | 1.6 | 46 | 46 |

[a] Reaction conditions: TiO_2_ anode, Ni foam cathode, 1 (0.1 mmol), 2 (0.3 mL), 5 mL acetone/water (V/V) = 19/1, HCl (100 μL), [Cu] (5% mol), Electrolyte (0.1 M), 40 °C, 13h. [b] Determined by ^1^H NMR analysis using 1,3,5-trimethoxybenzene as the internal standard.

Table S2. IMPS spectra of the fresh TiO_2_, the TiO_2_ after reaction without Cu^2+^and the TiO_2_ after reaction with Cu^2+^.

| Entry | k_ct_ | k_rec_ | η_ct_ |
| --- | --- | --- | --- |
| Fresh TiO_2_^[a]^ | 0.5250 | 0.1029 | 83.6% |
| Fresh TiO_2_^[b]^ | 1.1509 | 0.2306 | 83.3% |
| TiO_2_ after reaction without Cu^2+[a]^ | 1.5069 | 1.6404 | 47.9% |
| TiO_2_ after reaction with Cu^2+[a]^ | 1.2531 | 0.5115 | 71.0% |

[a] All were performed in standard reaction conditions without Cu^2+^. [b] In standard reaction conditions with Cu^2+^.

Table S3. Raman spectra of the fresh TiO_2_, the TiO_2_ after reaction without Cu^2+^ and with Cu^2+^.

|  | E_g_ area | A_1g_ area | Area ratio:$S_{A_{1g}}/S_{E_{g}}$ |
| --- | --- | --- | --- |
| Fresh TiO_2_ | 1329387.6 | 1119861.4 | 0.84 |
| TiO_2_ after reaction with Cu^2+^ | 1693696.6 | 1456718.3 | 0.86 |
| TiO_2_ after reaction without Cu^2+^ | 1700870.3 | 1755842.5 | 1.03 |

**Characterization data for compounds.**

**4-cyclohexyl-2-phenylquinoline (3A). ^1^H NMR** (400 MHz, Chloroform-*d*) δ 8.23 – 8.13 (m, 3H), 8.11 (d, *J* = 1.6 Hz, 1H), 7.76 (s, 1H), 7.73 – 7.67 (m, 1H), 7.53 (td, *J* = 7.5, 2.4 Hz, 3H), 7.46 (t, *J* = 7.2 Hz, 1H), 3.38 (tt, *J* = 11.5, 3.2 Hz, 1H), 2.09 (d, *J* = 10.4 Hz, 2H), 2.00 – 1.92 (m, 2H), 1.88 (dd, *J* = 13.3, 3.4 Hz, 1H), 1.70 – 1.54 (m, 4H), 1.41 (d, *J* = 11.2 Hz, 1H). **^13^C NMR** (101 MHz, Chloroform-*d*) δ 157.51, 154.09, 148.75, 140.45, 130.86, 129.25, 129.14, 128.91, 127.76, 126.02, 122.99, 115.65, 39.27, 33.82, 27.12, 26.47.

**4-cyclopentyl-2-phenylquinoline (4A). ^1^H NMR** (400 MHz, Chloroform-*d*) δ 8.19 (d, *J* = 8.6 Hz, 2H), 8.17 – 8.11 (m, 3H), 7.78 (s, 1H), 7.76 – 7.67 (m, 1H), 7.54 (d, *J* = 2.3 Hz, 3H), 7.46 (dd, *J* = 8.8, 5.5 Hz, 1H), 3.90 – 3.76 (m, 1H), 2.35 – 2.19 (m, 2H), 1.97 – 1.79 (m, 6H). **^13^C NMR** (101 MHz, Chloroform-*d*) δ 157.31, 152.82, 148.56, 140.31, 130.54, 129.11, 129.03, 128.84, 128.77, 127.62, 127.59, 126.77, 125.82, 123.56, 115.17, 40.84, 33.43, 25.53.

**4-cycloheptyl-2-phenylquinoline (5A). ^1^H NMR** (400 MHz, Chloroform-*d*) δ 8.19 (d, *J* = 8.3 Hz, 1H), 8.17 – 8.12 (m, 2H), 8.08 (d, *J* = 8.5 Hz, 1H), 7.76 (s, 1H), 7.70 (ddd, *J* = 8.3, 6.7, 1.4 Hz, 1H), 7.54 (dtd, *J* = 7.6, 5.6, 2.5 Hz, 3H), 7.46 (d, *J* = 7.0 Hz, 1H), 3.54 (tt, *J* = 10.6, 3.3 Hz, 1H), 2.19 – 2.07 (m, 2H), 1.99 – 1.78 (m, 6H), 1.76 – 1.67 (m, 4H). **^13^C NMR** (101 MHz, Chloroform-*d*) δ 157.47, 156.04, 148.79, 130.87, 129.24, 129.14, 128.91, 127.76, 126.03, 125.76, 123.09, 115.91, 40.87, 36.02, 28.02, 27.75.

**4-cyclooctyl-2-phenylquinoline (6A). ^1^H NMR** (400 MHz, Chloroform-*d*) δ 8.19 (d, *J* = 8.5 Hz, 1H), 8.17 – 8.12 (m, 2H), 8.09 (d, *J* = 8.4 Hz, 1H), 7.75 (s, 1H), 7.70 (t, *J* = 7.6 Hz, 1H), 7.59 – 7.49 (m, 3H), 7.46 (t, *J* = 7.2 Hz, 1H), 3.66 (tt, *J* = 8.8, 4.3 Hz, 1H), 2.02 (dtt, *J* = 12.9, 9.3, 4.0 Hz, 4H), 1.95 – 1.83 (m, 2H), 1.75 (d, *J* = 8.8 Hz, 8H). **^13^C NMR** (101 MHz, Chloroform-*d*) δ 157.40, 156.50, 148.88, 140.44, 130.91, 129.24, 129.11, 127.76, 126.03, 125.76, 123.20, 116.38, 33.92, 27.00, 26.74, 26.38.

**4-cyclododecyl-2-phenylquinoline (7A). ^1^H NMR** (400 MHz, Chloroform-*d*) δ 8.20 (d, *J* = 8.4 Hz, 1H), 8.13 (t, *J* = 7.4 Hz, 3H), 7.75 (s, 1H), 7.71 (t, *J* = 7.7 Hz, 1H), 7.54 (q, *J* = 7.1 Hz, 3H), 7.46 (t, *J* = 7.3 Hz, 1H), 3.71 (p, J = 6.5 Hz, 1H), 2.52 – 2.40 (m, 2H), 2.07 – 1.90 (m, 2H), 1.53 – 1.41 (m, 16H), 0.88 – 0.83 (m, 2H). **^13^C NMR** (101 MHz, Chloroform-*d*) δ 157.27, 154.02, 148.82, 140.47, 130.90, 129.25, 129.14, 128.91, 127.81, 126.70, 126.08, 123.03, 116.87, 40.56, 29.85, 24.25, 23.73, 23.68, 22.74.

**4-(7-oxabicyclo[2.2.1]heptan-2-yl)-2-phenylquinoline (8A). ^1^H NMR** (400 MHz, Chloroform-*d*) δ 8.24 – 8.13 (m, 3H), 8.04 (d, *J* = 8.5 Hz, 1H), 8.00 (s, 1H), 7.71 (t, *J* = 7.6 Hz, 1H), 7.53 (q, *J* = 8.1 Hz, 3H), 7.45 (t, *J* = 7.2 Hz, 1H), 4.86 – 4.80 (m, 2H), 3.72 (dd, *J* = 9.1, 4.7 Hz, 1H), 2.36 – 2.19 (m, 1H), 1.90 (dd, *J* = 11.3, 5.5 Hz, 2H), 1.75 – 1.55 (m, 4H).

**4-(1-ethoxyethyl)-2-phenylquinoline (9A). ^1^H NMR** (400 MHz, Chloroform-*d*) δ 8.21 (dd, *J* = 10.4, 8.3 Hz, 3H), 8.11 (d, *J* = 5.1 Hz, 1H), 8.00 (s, 1H), 7.76 – 7.68 (m, 1H), 7.54 (dd, *J* = 8.5, 6.6 Hz, 3H), 7.47 (t, *J* = 7.2 Hz, 1H), 5.20 (q, *J* = 6.6 Hz, 1H), 3.51 (q, *J* = 7.0 Hz, 2H), 1.65 (s, 3H), 1.28 (t, *J* = 7.1 Hz, 3H). **^13^C NMR** (101 MHz, Chloroform-d) δ 157.49, 150.50, 148.85, 139.97, 130.85, 129.46, 129.36, 128.97, 127.73, 126.25, 125.19, 123.04, 115.53, 74.76, 64.89, 23.63, 15.67.

**1-(2-phenylquinolin-4-yl)ethan-1-ol (10A). ^1^H NMR** (400 MHz, Chloroform-*d*) δ 8.19 (d, *J* = 8.4 Hz, 1H), 8.15 – 8.07 (m, 2H), 7.98 (s, 1H), 7.91 (dd, *J* = 8.4, 1.3 Hz, 1H), 7.70 (ddd, *J* = 8.4, 6.9, 1.4 Hz, 1H), 7.55 – 7.41 (m, 4H), 5.59 (q, *J* = 6.5 Hz, 1H), 2.42 (d, *J* = 85.4 Hz, 1H), 1.64 (d, *J* = 6.5 Hz, 3H). **^13^C NMR** (101 MHz, Chloroform-*d*) δ 157.32, 151.82, 148.36, 139.58, 130.53, 129.36, 129.25, 128.78, 127.52, 126.18, 124.28, 122.70, 114.40, 66.41, 24.58.

**4-methyl-2-phenylquinoline (11A). ^1^H NMR** (400 MHz, Chloroform-d) δ 8.16 (d, *J* = 1.8 Hz, 3H), 8.04 – 7.98 (m, 1H), 7.73 (d, *J* = 6.9 Hz, 2H), 7.60 – 7.50 (m, 3H), 7.50 – 7.43 (m, 1H), 2.78 (s, 3H). **^13^C NMR** (101 MHz, Chloroform-*d*) δ 157.24, 148.28, 144.96, 139.98, 130.45, 129.34, 128.92, 127.70, 127.60, 126.18, 123.75, 119.93, 19.16.

**4-(methyl-d3)-2-phenylquinoline (12A). ^1^H NMR** (400 MHz, Chloroform-*d*) δ 8.18 (dd, *J* = 8.1, 5.4 Hz, 3H), 7.86 (dd, *J* = 20.1, 8.4 Hz, 2H), 7.74 (t, *J* = 7.7 Hz, 1H), 7.58 – 7.51 (m, 3H), 7.47 (t, *J* = 7.3 Hz, 1H). **^13^C NMR** (101 MHz, Chloroform-*d*) δ 157.53, 148.45, 139.85, 136.92, 129.63, 129.47, 128.99, 127.73, 127.61, 127.34, 126.43, 119.16.

**4-(1,4-dioxan-2-yl)-2-phenylquinoline (13A). ^1^H NMR** (400 MHz, Chloroform-*d*) δ 8.24 – 8.17 (m, 3H), 8.10 (d, *J* = 3.2 Hz, 1H), 8.04 – 7.96 (m, 1H), 7.72 (s, 1H), 7.54 (q, *J* = 8.0 Hz, 3H), 7.50 – 7.41 (m, 1H), 5.42 (d, *J* = 10.0 Hz, 1H), 4.18 (d, *J* = 12.0 Hz, 1H), 4.09 (q, *J* = 3.5 Hz, 2H), 3.91 (d, *J* = 11.8 Hz, 1H), 3.84 (dd, *J* = 10.2, 4.9 Hz, 1H), 3.51 (td, *J* = 11.3, 3.2 Hz, 1H). **^13^C NMR** (101 MHz, Chloroform-*d*) δ 157.38, 148.29, 144.18, 139.62, 130.78, 129.42, 129.39, 128.82, 127.64, 126.53, 122.34, 116.20, 74.44, 72.13, 67.39, 66.67.

**2-phenyl-4-(tetrahydrofuran-2-yl)quinoline (14A)**. **^1^H NMR** (400 MHz, Chloroform-*d*) δ 8.21 (dd, *J* = 16.7, 8.0 Hz, 3H), 8.05 (s, 1H), 7.91 (d, *J* = 8.4 Hz, 1H), 7.72 (t, *J* = 7.8 Hz, 1H), 7.52 (d, *J* = 8.1 Hz, 3H), 7.46 (d, *J* = 7.4 Hz, 1H), 5.67 (t, *J* = 7.2 Hz, 1H), 4.28 (t, *J* = 7.1 Hz, 1H), 4.09 (q, *J* = 7.5 Hz, 1H), 2.67 (dq, *J* = 14.4, 7.6 Hz, 1H), 2.08 (ddq, *J* = 26.4, 12.5, 6.2 Hz, 2H), 1.91 (dq, *J* = 14.0, 7.0 Hz, 1H).

**4-(2-phenylquinolin-4-yl)butan-1-ol (15A).** **^1^H NMR** (400 MHz, Chloroform-*d*) δ 8.17 (dd, *J* = 13.6, 7.8 Hz, 3H), 8.05 (d, *J* = 8.3 Hz, 1H), 7.76 – 7.67 (m, 2H), 7.53 (t, *J* = 8.3 Hz, 3H), 7.46 (t, *J* = 7.2 Hz, 1H), 5.12 (dd, *J* = 4.5, 1.8 Hz, 1H), 3.94 – 3.81 (m, 2H), 3.75 (dt, *J* = 9.8, 6.5 Hz, 1H), 3.45 (dt, *J* = 9.8, 6.4 Hz, 1H), 3.16 (t, *J* = 7.8 Hz, 2H), 1.89 (ddd, *J* = 8.4, 6.4, 2.9 Hz, 4H), 1.74 (s, 2H). **^13^C NMR** (101 MHz, Chloroform-*d*) δ 157.24, 149.08, 148.68, 140.09, 130.68, 129.32, 128.91, 127.71, 126.68, 126.11, 123.53, 118.91, 104.06, 67.03, 32.50, 32.37, 27.05.

**2-phenyl-4-(tetrahydro-2H-pyran-2-yl)quinoline (16A). ^1^H NMR** (400 MHz, Chloroform-*d*) δ 8.20 (d, *J* = 7.9 Hz, 3H), 8.06 (s, 1H), 7.99 (d, *J* = 8.4 Hz, 1H), 7.70 (t, *J* = 7.6 Hz, 1H), 7.52 (t, *J* = 7.6 Hz, 3H), 7.45 (t, *J* = 7.2 Hz, 1H), 5.09 (dd, *J* = 11.2, 2.0 Hz, 1H), 4.37 – 4.23 (m, 1H), 3.79 (td, J = 11.6, 2.7 Hz, 1H), 2.12 (d, *J* = 13.3 Hz, 1H), 2.03 (d, *J* = 7.5 Hz, 1H), 1.84 (td, *J* = 11.5, 10.0, 4.4 Hz, 2H), 1.73 – 1.64 (m, 2H). **^13^C NMR** (101 MHz, Chloroform-*d*) δ 157.59, 149.33, 148.61, 140.08, 130.82, 129.35, 129.18, 128.86, 127.80, 126.14, 124.47, 122.99, 115.53, 76.55, 69.43, 33.91, 26.12, 24.25.

**4-(3,5-dimethylbenzyl)-2-phenylquinoline (17A). ^1^H NMR** (400 MHz, Chloroform-*d*) δ 8.24 (s, 1H), 8.14 (d, J = 7.3 Hz, 2H), 8.03 (d, *J* = 8.3 Hz, 1H), 7.71 (d, *J* = 17.7 Hz, 2H), 7.50 – 7.46 (m, 4H), 6.88 (s, 1H), 6.85 (s, 2H), 4.42 (s, 2H), 2.27 (s, 6H). **^13^C NMR** (101 MHz, Chloroform-*d*) δ 157.52, 148.46, 139.86, 138.37, 136.90, 130.55, 129.45, 129.38, 128.98, 128.42, 127.60, 127.34, 126.77, 126.42, 124.00, 119.14, 38.59, 21.42.

**4-(4-methylbenzyl)-2-phenylquinoline (18A). ^1^H NMR** (400 MHz, Chloroform-*d*) δ 8.18 (d, *J* = 1.4 Hz, 1H), 8.13 – 8.08 (m, 2H), 8.05 – 7.98 (m, 1H), 7.75 – 7.69 (m, 1H), 7.65 (s, 1H), 7.55 – 7.43 (m, 5H), 7.12 (s, 4H), 4.46 (s, 2H), 2.32 (s, 3H). **^13^C NMR** (101 MHz, Chloroform-*d*) δ 157.20, 148.60, 147.33, 136.17, 135.70, 130.48, 129.43, 129.31, 129.23, 128.85, 128.74, 127.58, 126.65, 126.29, 123.78, 119.84, 38.17, 21.04.

**2-cyclohexylquinoline (19A). ^1^H NMR** (400 MHz, Chloroform-*d*) δ 8.06 (dd, *J* = 12.3, 8.5 Hz, 2H), 7.77 (d, *J* = 8.1 Hz, 1H), 7.67 (t, *J* = 7.7 Hz, 1H), 7.47 (t, *J* = 7.5 Hz, 1H), 7.33 (d, *J* = 8.5 Hz, 1H), 2.93 (tt, *J* = 12.0, 3.5 Hz, 1H), 2.02 (t, *J* = 6.3 Hz, 2H), 1.97 – 1.85 (m, 2H), 1.83 – 1.76 (m, 1H), 1.65 (d, *J* = 3.3 Hz, 2H), 1.54 – 1.44 (m, 2H), 1.38 – 1.21 (m, 1H). ^13^C NMR (101 MHz, Chloroform-*d*) δ 166.99, 148.02, 136.42, 129.35, 129.19, 127.58, 125.74, 119.74, 47.80, 33.01, 26.75, 26.28.

**4-cyclohexylquinoline (19B).** ^1^H NMR (400 MHz, Chloroform-*d*) δ 8.85 (d, *J* = 4.6 Hz, 1H), 8.20 – 8.05 (m, 2H), 7.70 (ddd, *J* = 8.3, 6.7, 1.4 Hz, 1H), 7.61 – 7.51 (m, 1H), 3.41 – 3.27 (m, 1H), 2.04 (d, *J* = 26.3 Hz, 2H), 1.95 (dd, *J* = 8.0, 5.0 Hz, 2H), 1.90 – 1.82 (m, 1H), 1.56 (d, *J* = 2.9 Hz, 5H), 1.42 (m, 1H).

**1-cyclohexylisoquinoline (20A). ^1^H NMR** (400 MHz, Chloroform-*d*) δ 8.48 (d, *J* = 5.7 Hz, 1H), 8.23 (d, *J* = 8.5 Hz, 1H), 7.81 (d, *J* = 8.2 Hz, 1H), 7.69 – 7.56 (m, 2H), 7.48 (d, *J* = 5.7 Hz, 1H), 3.65 – 3.50 (m, 1H), 2.06 – 1.88 (m, 4H), 1.90 – 1.77 (m, 3H), 1.62 – 1.49 (m, 2H), 1.47 – 1.37 (m, 1H). **^13^C NMR** (101 MHz, Chloroform-*d*) δ 165.87, 142.10, 136.56, 129.67, 127.70, 126.94, 126.45, 124.88, 119.01, 41.70, 32.73, 27.04, 26.40.

**6-cyclohexylphenanthridine (21A).** **^1^H NMR** (400 MHz, Chloroform-*d*) δ 8.64 (d, *J* = 8.3 Hz, 1H), 8.56 – 8.51 (m, 1H), 8.32 (d, *J* = 8.2 Hz, 1H), 8.17 (dd, *J* = 8.1, 1.3 Hz, 1H), 7.86 – 7.78 (m, 1H), 7.74 – 7.66 (m, 2H), 7.63 – 7.57 (m, 1H), 3.67 – 3.57 (m, 1H), 2.15 – 2.05 (m, 2H), 2.02 – 1.91 (m, 4H), 1.86 (dd, *J* = 12.3, 3.4 Hz, 1H), 1.60 (d, *J* = 3.3 Hz, 2H), 1.48 (dd, *J* = 12.3, 6.3 Hz, 1H). **^13^C NMR** (101 MHz, Chloroform-*d*) δ 165.45, 143.76, 133.21, 129.88, 128.57, 127.24, 126.34, 125.80, 124.79, 123.48, 122.71, 121.94, 42.12, 32.39, 26.99, 26.41.

**4-cyclohexyl-2-methylquinoline (22A).** **^1^H NMR** (400 MHz, Chloroform-*d*) δ 8.04 (dd, *J* = 8.5, 5.8 Hz, 2H), 7.66 (dt, *J* = 15.3, 7.7 Hz, 1H), 7.52 – 7.44 (m, 1H), 7.17 (s, 1H), 3.29 (tt, *J* = 8.9, 3.3 Hz, 1H), 2.72 (s, 3H), 2.00 (d, *J* = 8.9 Hz, 2H), 1.97 – 1.90 (m, 2H), 1.89 – 1.80 (m, 1H), 1.53 (pd, *J* = 12.9, 11.1, 4.2 Hz, 4H), 1.36 (s, 1H). **^13^C NMR** (101 MHz, Chloroform-*d*) δ 158.93, 153.53, 148.21, 129.62, 129.56, 125.42, 125.30, 122.97, 118.46, 38.94, 33.71, 27.08, 26.47, 25.62.

**2-cyclohexyl-4-methylquinoline (26A). ^1^H NMR** (400 MHz, Chloroform-*d*) δ 8.07 – 8.02 (m, 1H), 7.94 (dd, *J* = 8.4, 1.5 Hz, 1H), 7.69 – 7.63 (m, 1H), 7.53 – 7.46 (m, 1H), 7.17 (d, *J* = 1.3 Hz, 1H), 2.94 – 2.83 (m, 1H), 2.68 (s, 3H), 2.06 – 1.98 (m, 2H), 1.89 (dt, *J* = 12.7, 3.3 Hz, 2H), 1.84 – 1.76 (m, 1H), 1.70 – 1.57 (m, 2H), 1.53 – 1.43 (m, 2H), 1.41 – 1.30 (m, 1H). **^13^C NMR** (101 MHz, Chloroform-*d*) δ 166.54, 144.27, 129.51, 128.95, 127.07, 125.38, 123.57, 120.27, 77.23, 47.61, 32.85, 26.58, 26.15, 18.84.

**4-bromo-1-cyclohexylisoquinoline (27A).** **^1^H NMR** (400 MHz, Chloroform-*d*) δ 8.52 (s, 1H), 8.27 – 8.19 (m, 2H), 7.78 (t, *J* = 7.6 Hz, 1H), 7.65 (t, *J* = 7.7 Hz, 1H), 3.52 (tt, *J* = 11.7, 3.2 Hz, 1H), 1.94 (ddd, *J* = 13.2, 9.2, 3.5 Hz, 4H), 1.82 – 1.70 (m, 2H), 1.60 – 1.44 (m, 3H), 1.40 – 1.29 (m, 2H). **^13^C NMR** (101 MHz, Chloroform-*d*) δ 164.87, 140.89, 133.94, 129.64, 127.75, 127.30, 125.19, 124.34, 41.65, 32.72, 32.69, 26.94, 26.32.

**4,7-dichloro-2-cyclohexylquinoline (28A). ^1^H NMR** (400 MHz, Chloroform-*d*) δ 8.08 (dd, J = 13.0, 2.3 Hz, 2H), 7.51 (dd, J = 8.9, 2.1 Hz, 1H), 7.40 (s, 1H), 2.92 – 2.80 (m, 1H), 2.01 (ddt, J = 12.1, 3.6, 1.8 Hz, 2H), 1.90 (dq, J = 12.8, 3.3 Hz, 2H), 1.84 – 1.72 (m, 1H), 1.67 – 1.54 (m,2H), 1.53 – 1.40 (m, 2H), 1.37 – 1.30 (m, 1H). **^13^C NMR** (101 MHz, Chloroform-*d*) δ 168.17, 149.13, 142.53, 136.21, 128.39, 127.55, 125.33, 123.65, 120.16, 47.32, 32.57, 26.39, 25.97.


**6-cyclohexylpyridine-2,4-dicarbonitrile (29A). ^1^H NMR** (400 MHz, Chloroform-*d*) δ 8.81 (s, 1H), 7.83 (s, 1H), 3.07 – 2.94 (m, 1H), 1.94 (d, *J* = 10.5 Hz, 4H), 1.84 (d, *J* = 13.5 Hz, 1H), 1.61 – 1.46 (m, 4H), 1.36 – 1.30 (m, 1H). **^13^C NMR** (101 MHz, Chloroform-*d*) δ 150.97, 148.55, 132.18, 129.87, 121.04, 115.80, 114.08, 41.80, 32.94, 26.24, 25.44.

**3-Cyclohexylbenzo[b]thiophene-2-carbaldehyde (31A). ^1^H NMR** (400 MHz, Chloroform-*d*) δ 10.49 (s, 1H), 8.08 (d, *J* = 8.2 Hz, 1H), 7.86 (d, *J* = 8.0 Hz, 1H), 7.46 (d, *J* = 7.7 Hz, 1H), 7.40 (t, *J* = 7.6 Hz, 1H), 3.54 (p, *J* = 7.9 Hz, 1H), 2.07 – 1.92 (m, 6H), 1.88 (dq, *J* = 11.6, 3.1 Hz, 1H), 1.55 – 1.38 (m, 3H). **^13^C NMR** (101 MHz, Chloroform-*d*) δ 184.33, 152.15, 142.88, 139.00, 138.10, 127.84, 125.65, 124.37, 123.54, 39.50, 33.64, 27.10, 26.04.

**5-Bromo-2-cyclohexylbenzo[d]thiazole (32A). ^1^H NMR** (400 MHz, Chloroform-*d*) δ 8.11 (s, 1H), 7.70 (d, *J* = 8.4 Hz, 1H), 7.45 (d, *J* = 8.5 Hz, 1H), 3.09 (tt, *J* = 11.7, 3.7 Hz, 1H), 2.28 – 2.14 (m, 2H), 1.89 (dt, *J* = 12.9, 3.6 Hz, 2H), 1.85 – 1.52 (m, 6H).

**3-chloro-4-cyclohexyl-6-phenylpyridazine (35A). ^1^H NMR** (400 MHz, Chloroform-*d*) δ 8.03 (d, *J* = 6.9 Hz, 2H), 7.67 (s, 1H), 7.52 (d, *J* = 6.4 Hz, 3H), 2.95 (t, *J* = 11.5 Hz, 1H), 2.01 (d, *J* = 11.6 Hz, 2H), 1.93 (d, *J* = 13.2 Hz, 2H), 1.84 (d, *J* = 13.4 Hz, 1H), 1.50 (d, *J* = 13.7 Hz, 4H), 1.42-1.39 (m, 1H).

**2,6-dichloro-8-cyclohexyl-9-methyl-9H-purine (36A). ^1^H NMR** (400 MHz, Chloroform-*d*) δ 3.81 (s, 3H), 2.93 – 2.80 (m, 1H), 2.02 – 1.90 (m, 4H), 1.86 (dd, *J* = 12.0, 3.1 Hz, 4H), 1.45 – 1.36 (m, 3H). **^13^C NMR** (101 MHz, Chloroform-*d*) δ 162.84, 154.47, 151.46, 149.34, 129.24, 36.67, 30.52, 28.74, 25.69, 25.22.

**3,4-bis(chloromethyl)-1-tosylpyrrolidine (41A). ^1^H NMR** (400 MHz, Chloroform-*d*) δ 7.72 (t, *J* = 7.7 Hz, 2H), 7.35 (dd, *J* = 8.3, 2.5 Hz, 2H), 3.45 (ddt, *J* = 20.2, 9.6, 5.8 Hz, 4H), 3.36 – 3.04 (m, 3H), 2.63 (dq, *J* = 11.8, 6.4 Hz, 1H), 2.46 – 2.25 (m, 4H). **^13^C NMR** (101 MHz, Chloroform-*d*) δ 144.01, 133.51, 132.66, 129.97, 127.92, 127.67, 51.25, 50.68, 45.27, 43.95, 43.34, 41.96, 21.70.

**NMR Spectra for New Compounds**

Compound 3A

^1^H NMR (400 MHz, CDCl_3_) of 3A

^13^C NMR (101 MHz MHz, CDCl_3_) of 3A

Compound 4A

^1^H NMR (400 MHz, CDCl_3_) of 4A

^13^C NMR (101 MHz MHz, CDCl_3_) of 4A

Compound 5A

^1^H NMR (400 MHz, CDCl_3_) of 5A

^13^C NMR (101 MHz MHz, CDCl_3_) of 5A

Compound 6A

^1^H NMR (400 MHz, CDCl_3_) of 6A

^13^C NMR (101 MHz MHz, CDCl_3_) of 6A

Compound 7A

^1^H NMR (400 MHz, CDCl_3_) of 7A

^13^C NMR (101 MHz MHz, CDCl_3_) of 7A

Compound 8A

^1^H NMR (400 MHz, CDCl_3_) of 8A

Compound 9A

^1^H NMR (400 MHz, CDCl_3_) of 9A

^13^C NMR (101 MHz MHz, CDCl_3_) of 9A

Compound 10A

^1^H NMR (400 MHz, CDCl_3_) of 10A，* impurity

^13^C NMR (101 MHz MHz, CDCl_3_) of 10A

Compound 11A

^1^H NMR (400 MHz, CDCl_3_) of 11A

^13^C NMR (101 MHz MHz, CDCl_3_) of 11A

Compound 12A

^1^H NMR (400 MHz, CDCl_3_) of 12A

^13^C NMR (101 MHz MHz, CDCl_3_) of 12A

^2^H NMR (600 MHz, CDCl_3_) of 12A

Compound 13A

^1^H NMR (400 MHz, CDCl_3_) of 13A

^13^C NMR (101 MHz MHz, CDCl_3_) of 13A

Compound 14A

^1^H NMR (400 MHz, CDCl_3_) of 14A

Compound 15A

^1^H NMR (400 MHz, CDCl_3)_ of 15A

^13^C NMR (101 MHz MHz, CDCl_3_) of 15A

Compound 16A

^1^H NMR (400 MHz, CDCl_3_) of 16A

^13^C NMR (101 MHz MHz, CDCl_3_) of 16A

Compound 17A

^1^H NMR (400 MHz, CDCl_3_) of 17A


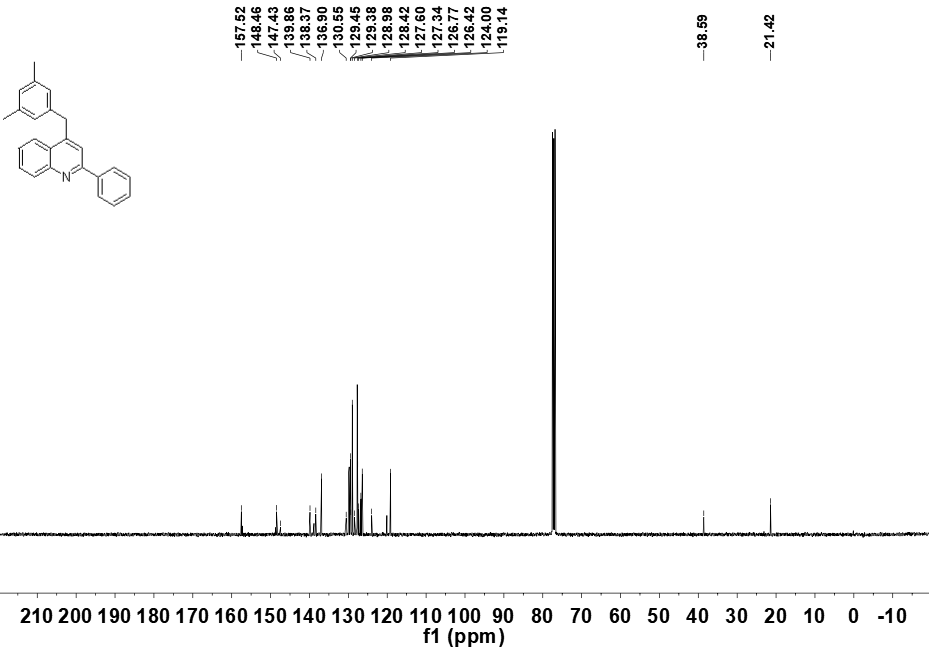


^13^C NMR (101 MHz MHz, CDCl_3_) of 17A

Compound 18A

^1^H NMR (400 MHz, CDCl_3_) of 18A

^13^C NMR (101 MHz MHz, CDCl_3_) of 18A

Compound 19A

^1^H NMR (400 MHz, CDCl_3_) of 19A

^13^C NMR (101 MHz MHz, CDCl_3_) of 19A

Compound 19B

^1^H NMR (400 MHz, CDCl_3_) of 19B

Compound 20A

^1^H NMR (400 MHz, CDCl_3_) of 20A

^13^C NMR (101 MHz MHz, CDCl_3_) of 20A

Compound 21A

^1^H NMR (400 MHz, CDCl_3_) of 21A

^13^C NMR (101 MHz MHz, CDCl_3_) of 21A

Compound 22A

^1^H NMR (400 MHz, CDCl_3_) of 22A

^13^C NMR (101 MHz MHz, CDCl_3_) of 22A

Compound 26A

^1^H NMR (400 MHz, CDCl_3_) of 26A

^13^C NMR (101 MHz MHz, CDCl_3_) of 26A

Compound 27A

^1^H NMR (400 MHz, CDCl_3_) of 27A

^13^C NMR (101 MHz MHz, CDCl_3_) of 27A

Compound 28A

^1^H NMR (400 MHz, CDCl_3_) of 28A

^13^C NMR (101 MHz MHz, CDCl_3_) of 28A

Compound 29A

^1^H NMR (400 MHz, CDCl_3_) of 29A

^13^C NMR (101 MHz MHz, CDCl_3_) of 29A

Compound 31A

^1^H NMR (400 MHz, CDCl_3_) of 31A

^13^C NMR (101 MHz MHz, CDCl_3_) of 31A

Compound 32A

^1^H NMR (400 MHz, CDCl_3_) of 32A

Compound 35A

^1^H NMR (400 MHz, CDCl_3_) of 35A

Compound 36A

^1^H NMR (400 MHz, CDCl_3_) of 36A

^13^C NMR (101 MHz MHz, CDCl_3_) of 36A

Compound 41A

^1^H NMR (400 MHz, CDCl_3_) of 41A

^13^C NMR (101 MHz MHz, CDCl_3_) of 41A

**Reference**

1. Xue, J.; Wu, L.; Deng, C.; Tang, D.; Wang, S.; Ji, H.; Chen, C.; Zhang, Y.; Zhao, J., Plasmon-Mediated Electrochemical Activation of Au/TiO_2_ Nanostructure-Based Photoanodes for Enhancing Water Oxidation and Antibiotic Degradation. *ACS Appl. Nano Mater.* **2022,** *5*, 11342-11351.

2. Wu, L.; Li, Q.; Dang, K.; Tang, D.; Chen, C.; Zhang, Y.; Zhao, J., Highly Selective Ammonia Oxidation on BiVO_4_ Photoanodes Co‐catalyzed by Trace Amounts of Copper Ions. *Angew. Chem. Int. Ed.* **2024,** *136*, e202316218.

3. Wu, L.; Tang, D.; Xue, J.; Liu, S.; Wang, J.; Ji, H.; Chen, C.; Zhang, Y.; Zhao, J., Competitive Non‐Radical Nucleophilic Attack Pathways for NH_3_ Oxidation and H_2_O Oxidation on Hematite Photoanodes. *Angew. Chem. Int. Ed.* **2022,** *61*, e202214580.

4. VandeVondele, J.; Krack, M.; Mohamed, F.; Parrinello, M.; Chassaing, T.; Hutter, J., Quickstep: Fast and accurate density functional calculations using a mixed Gaussian and plane waves approach. *Comput. Phys. Commun.* **2005,** *167*, 103-128.

5. Hutter, J.; Iannuzzi, M.; Schiffmann, F.; VandeVondele, J., cp2k: atomistic simulations of condensed matter systems. *WIREs Comput. Mol. Sci.* **2013,** *4*, 15-25.

6. Grimme, S.; Ehrlich, S.; Goerigk, L., Effect of the damping function in dispersion corrected density functional theory. *J. Comput. Chem.* **2011,** *32*, 1456-1465.

7. Treacy, S. M.; Rovis, T., Copper Catalyzed C(sp^3^)–H Bond Alkylation via Photoinduced Ligand-to-Metal Charge Transfer. *J. Am. Chem. Soc.* **2021,** *143*, 2729-2735.

8. Lu, T.; Chen, Q., Shermo: A general code for calculating molecular thermochemistry properties. *Comput. Theor. Chem* **2021,** *1200*, 113249.

9. Huang, C.-Y.; Li, J.; Li, C.-J., A cross-dehydrogenative C(sp^3^)−H heteroarylation via photo-induced catalytic chlorine radical generation. *Nat. Commun.* **2021,** *12*, 4010.

10. Fu, N.; Sauer, G. S.; Lin, S., Electrocatalytic Radical Dichlorination of Alkenes with Nucleophilic Chlorine Sources. *J. Am. Chem. Soc.* **2017,** *139*, 15548-15553.

11. Xu, P.; Chen, P. Y.; Xu, H. C., Scalable Photoelectrochemical Dehydrogenative Cross‐Coupling of Heteroarenes with Aliphatic C−H Bonds. *Angew. Chem. Int. Ed.* **2020,** *59*, 14275-14280.

12. Tang, D.; Wu, L.; Li, L.; Fu, N.; Chen, C.; Zhang, Y.; Zhao, J., A controlled non-radical chlorine activation pathway on hematite photoanodes for efficient oxidative chlorination reactions. *Chem. Sci.* **2024,** *15*, 3018-3027.

13. Li, Z.; Luo, L.; Li, M.; Chen, W.; Liu, Y.; Yang, J.; Xu, S.-M.; Zhou, H.; Ma, L.; Xu, M.; Kong, X.; Duan, H., Photoelectrocatalytic C–H halogenation over an oxygen vacancy-rich TiO_2_ photoanode. *Nat. Commun.* **2021,** *12*, 6698.

14. Cao, X.; Chen, Z.; Lin, R.; Cheong, W.-C.; Liu, S.; Zhang, J.; Peng, Q.; Chen, C.; Han, T.; Tong, X.; Wang, Y.; Shen, R.; Zhu, W.; Wang, D.; Li, Y., A photochromic composite with enhanced carrier separation for the photocatalytic activation of benzylic C–H bonds in toluene. *Nat. Catal.* **2018,** *1*, 704-710.

15. Zhu, K.; Zhu, Q.; Jiang, M.; Zhang, Y.; Shao, Z.; Geng, Z.; Wang, X.; Zeng, H.; Wu, X.; Zhang, W.; Huang, K.; Feng, S., Modulating Ti t_2g_ Orbital Occupancy in a Cu/TiO_2_ Composite for Selective Photocatalytic CO_2_ Reduction to CO. *Angew. Chem. Int. Ed.* **2022,** *61*, e202207600.

16. Paliwal, A.; Bandas, C. D.; Thornburg, E. S.; Haasch, R. T.; Gewirth, A. A., Enhanced Nitrate Reduction Activity from Cu-Alloy Electrodes in an Alkaline Electrolyte. *ACS Catal.* **2023,** *13*, 6754-6762.
